# *Limosilactobacillus reuteri* orchestrates IAA-mediated mitochondrial homeostasis and stem cell renewal against intestinal oxidative injury in aged hens

**DOI:** 10.1186/s40104-026-01499-4

**Published:** 2026-09-14

**Authors:** Shenao Zhan, Ziyu Wang, Yujie Lv, Weichen Huang, Chaoyue Ge, Lianchi Wu, Dongyou Yu, Bing Liu

**Affiliations:** 1 College of Animal Sciences, Zhejiang University, Hangzhou, 310058 China; 2 Hainan Institute, Zhejiang University, Sanya, 572000 China; 3 ZJU-Xinchang Joint Innovation Centre (TianMu Laboratory), Gaochuang Hi-Tech Park, Xinchang, 312500 China

**Keywords:** Intestinal barrier, Laying hens, *Limosilactobacillus reuteri*, Mitochondria, Oxidative stress

## Abstract

**Background:**

Intestinal oxidative stress compromises gut health and production performance in laying hens. Although *Limosilactobacillus reuteri* (*L. reuteri*) has been shown to mitigate oxidative stress, the underlying mechanisms remain incompletely understood. This study therefore investigated the protective effects and mechanisms of *L. reuteri* against diquat-induced intestinal oxidative injury in aged hens.

**Methods:**

A total of 270 70-week-old Jinbai hens were divided into control (CON), diquat (DQ), and *L. reuteri* + diquat (LRD) groups with 6 replicates each. Birds in CON and DQ groups received a basal diet, while those in LRD group received basal diet supplemented with *L. reuteri*. After a 10-week pretreatment, DQ and LRD hens were intraperitoneally injected with diquat, while CON hens were injected intraperitoneally with an equivalent amount of saline solution.

**Results:**

Diquat challenge significantly compromised intestinal barrier integrity, as evidenced the downregulated mRNA and protein expression of MUC2, ZO-1 and Occludin (*P* < 0.05). Notably, these adverse impacts were markedly reversed by *L. reuteri* pretreatment. Moreover, *L. reuteri* reshaped the gut microbiota by increasing the abundance of *Lactobacillus* (*P* < 0.05). Metabolomic analysis revealed significant enrichment of tryptophan metabolism, with a notable upregulation of indole-3-acetic acid (IAA). At the mitochondrial level, *L. reuteri* reduced mitochondrial ROS production, restored ATP content (*P* < 0.05), and downregulated mitochondrial biogenesis-related genes expression of *PGC-1α* and *NRF1* and balanced mitochondrial dynamics genes expression of *MFN1* and *DRP1* (*P* < 0.05). Furthermore, *L. reuteri* elevated GSH-Px and T-AOC activities via activation of the Nrf2 pathway, and reduced IL-1β and IL-6 levels by suppression of NF-κB signaling (*P* < 0.05). In vitro organoid culture further demonstrated IAA significantly reduced mitochondrial ROS production and restored ATP levels (*P* < 0.05) and stimulated stem cell proliferation. These changes inhibited apoptosis and promoted intestinal proliferation, thereby alleviating intestinal oxidative injury.

**Conclusion:**

*L. reuteri* alleviates intestinal oxidative damage via modulation of the gut microbiota–IAA axis, which coordinates mitochondrial homeostasis and stem cell renewal, thereby mitigating oxidative stress and enhancing barrier function. These findings highlight its potential as a probiotic therapeutic agent for mitigating oxidative stress and preserving gut health in aging poultry.

**Supplementary Information:**

The online version contains supplementary material available at https://doi.org/10.1186/s40104-026-01499-4.

## Introduction

Oxidative stress is a major challenge in modern poultry production, arising from high-density housing, heat stress, dietary imbalances, and pathogen exposure [1]. In laying hens, it occurs most prominently during the post-peak laying period due to sustained physiological demands and depleted antioxidant reserves [2, 3]. Excessive reactive oxygen species (ROS) cause oxidative damage to lipids, proteins, and DNA in intestinal epithelial cells, activating NF-κB signaling and triggering inflammation [4, 5]. This cascade leads to epithelial apoptosis, disruption of tight junction proteins, impaired epithelial renewal, and compromised intestinal barrier integrity [6, 7]. The consequent increase in intestinal permeability impairs calcium and phosphorus absorption, ultimately manifesting as reduced laying rate, deteriorated eggshell quality, poor feed efficiency, and increased disease susceptibility [7, 8]. Therefore, maintaining intestinal health in aging laying hens is critically important for sustaining productive performance.

Beyond direct cellular damage, oxidative stress also perturbs the gut microbiota and its associated metabolism [9]. Excessive ROS suppresses beneficial anaerobes, particularly *Lactobacillus* species, while promoting the expansion of pathobionts, leading to reduced microbial diversity and dysbiosis [9, 10]. This microbial imbalance disrupts tryptophan metabolism, shifting it away from the indole pathway and substantially diminishing the production of key metabolites such as indole-3-acetic acid (IAA) and indole-3-propionic acid (IPA) [11]. Under normal conditions, these indole derivatives normally activate the aryl hydrocarbon receptor (AhR), which upregulates tight junction proteins, promotes mucin secretion, and strengthens mucosal immunity [12, 13]. Their depletion thus weakens intestinal barrier function and removes important mitochondrial protective signals, as IAA and IPA are known to maintain mitochondrial integrity and suppress ROS generation [14]. Thus, microbiota dysbiosis coupled with tryptophan metabolic reprogramming exacerbates oxidative stress-induced intestinal damage.

Mitochondria play a central role in oxidative stress-induced intestinal injury, functioning both as the primary source and a principal target of ROS [15]. Under physiological conditions, the mitochondrial electron transport chain (ETC) generates controlled amounts of ROS as byproducts of oxidative phosphorylation, which are efficiently neutralized by mitochondrial antioxidant systems [16]. However, under conditions of excessive oxidative challenge, ROS overwhelm mitochondrial antioxidant defenses, provoking mitochondrial dysfunction characterized by collapsed membrane potential, impaired ETC complex activity, reduced ATP synthesis, decreased mtDNA copy number [17], dysregulated mitochondrial biogenesis and dynamics, and defective mitophagy [18, 19]. Critically, damaged mitochondria become amplifiers rather than controllers of oxidative stress: dysfunctional ETC complexes generate excessive mitochondrial ROS [20]. This mitochondrial ROS surplus suppresses Nrf2-mediated antioxidant signaling while concurrently activating NF-κB-driven inflammatory cascades, further exacerbating epithelial apoptosis, inhibiting crypt cell proliferation, and accelerating the breakdown of intestinal barrier integrity [21, 22]. Therefore, restoring mitochondrial functional homeostasis represents a pivotal therapeutic strategy for alleviating oxidative stress induced intestinal injury in laying hens.

Probiotic *Lactobacillus* strains have emerged as promising candidates for modulating mitochondrial function and intestinal oxidative stress [23–25]. Accumulating evidence demonstrates that *Lactobacillus* supplementation enhances mitochondrial biogenesis through PGC-1α/NRF1 activation [26], restores mitochondrial dynamic balance between fission and fusion [27], and optimizes mitophagy flux to selectively eliminate damaged mitochondria [28], collectively maintaining mitochondrial quality and function under oxidative challenge [29, 30]. *Limosilactobacillus reuteri*, a well-characterized *Lactobacillus* species, exerts these protective effects partly through its capacity to remodel gut microbiota composition and reprogram tryptophan metabolism toward the production of indole derivatives, which activate AhR signaling to reinforce intestinal barrier function and modulate immune homeostasis [31, 32].

Our laboratory isolated *L. reuteri* Y067 from the intestinal contents of healthy laying hens. As a host-adapted probiotic, it possesses better colonization capacity in the avian gut [33]. Our study in vivo showed that *L. reuteri* Y067 supplementation significantly improved production performance in aged hens. However, whether *L. reuteri*-driven microbiota remodeling and tryptophan metabolite reprogramming regulate mitochondrial function and promote intestinal barrier protection under oxidative stress remains unclear, particularly in laying hens. To address this gap, we established a diquat-induced intestinal oxidative stress model in laying hens to investigate whether and how *L. reuteri* Y067 alleviates intestinal injury, with a specific focus on its potential regulation of gut microbiota, tryptophan metabolism, mitochondrial function, and barrier integrity. By integrating microbiome profiling, metabolomics, and intestinal organoid validation, this study aims to elucidate the mechanisms underlying its protective effects on mitochondrial function and intestinal barrier integrity.

## Methods

### Experimental design

All procedures of the animal experiments were approved by the Animal Care and Use Committee of Zhejiang University (Hangzhou, China; approval number ZJU20241152). A total of 270 Jinbai laying hens (70 weeks old, laying rate 82.2% ± 1.2%) were randomly allocated to three groups, each with six replicates of 15 hens. The hens were commercially purchased from Tianjin Guanglong Biotechnology Co., Ltd. (Tianjin, China) and raised in an environmentally controlled house at the Huajie Poultry Company (Hangzhou, China). Randomization was stratified by body weight and pre-trial laying rate to ensure similar baseline characteristics across groups. Birds in control group (CON) and diquat-challenged group (DQ) received basal diets, while birds in *L. reuteri* supplementation with diquat-challenged group (LRD) received a basal diet supplemented with *L. reuteri* Y067 (2.0 × 10^8^ CFU/kg feed) for 10 weeks. The basal diets (Table 1) were formulated to meet the nutrient requirements recommended by the National Research Council [34]. *L. reuteri* Y067 was isolated and maintained in our laboratory and deposited in the China Center for Type Culture Collection (CCTCC NO: M 2024259). Our in vitro study showed *L. reuteri* Y067 exhibited superior antioxidant and anti-inflammatory activities (Supplementary material 2). After 10 weeks of dietary intervention, hens in the DQ and LRD groups received an intraperitoneal injection of diquat (1 mL/kg body weight, 10 mg/mL in 0.9% saline) to induce oxidative stress, as previously described [35, 36]. The CON group received an equivalent volume of 0.9% saline. Birds had unrestricted access to fresh water and mashed diets. The housing environment was maintained at 24 °C, 50%–60% humidity, with a 16-h light/8-h dark cycle.

**Table 1:** Ingredients and nutrient contents of the basal diet

**Ingredients, %**	**Contents**	**Nutrient levels**^**2**^	**Contents**
Corn (7.8% CP)	56	Metabolic energy, Mcal/kg	2.65
Soybean meal (44% CP)	25.5	Crude protein, %	16.5
Wheat middling (14.5% CP)	4	Lysine, %	0.84
Emulsified fat powder (50% Fat)	2	Methionine, %	0.48
Limestone	9	Cysteine + methionine, %	0.76
CaHPO_4_	1	Calcium, %	3.51
Salt	0.3	Total phosphorus, %	0.67
DL-Methionine	0.2	Available phosphorus, %	0.4
Premix^1^	2		
Total	100		

^1^The premix provided the following per kg of the diet: vitamin A, 12,500 IU; vitamin D_3_, 4,000 IU; vitamin E, 80 IU; vitamin K_3_, 2 mg; vitamin B_12_, 5 mg; thiamine, 1 mg; riboflavin, 8.5 mg; calcium pantothenate, 50 mg; niacin acid, 32.5 mg; pyridoxine, 8 mg; folic acid, 5 mg; iron, 60 mg; copper, 10 mg; manganese, 80 mg; zinc 80 mg; selenium 0.30 mg; iodine 0.3 mg; and antioxidant, 2.00 mg

^2^Metabolizable energy and available phosphorus contents were calculated values based on the Tables of Feed Composition and Nutritive Values in China (in Chinese, 32^th^ edition, 2021), while the others were measured values

### Sample collection

Seven days after diquat injection, one hen per replicate was randomly selected for sampling and subsequent analyses (*n* = 6). Blood was drawn from the wing vein, centrifuged at 4,000 r/min for 15 min at 4 °C, and serum was stored at −20 °C, as previously described [37]. Jejunal segments were fixed in 4% paraformaldehyde, while another mid‑jejunum sample was fixed in 2.5% glutaraldehyde. Additional mid‑jejunum tissue (∼10 cm) was snap‑frozen in liquid nitrogen and stored at −80 °C for molecular and biochemical analyses. Cecal contents were collected and stored under the same conditions for microbiota and metabolomic analyses.

### Laying performance

For laying performance analysis, the number of eggs and total egg weight were monitored daily, and feed disappearance was recorded weekly on a replicate basis (*n* = 6) to calculate the laying rate (LR), average daily egg mass (ADEM), average daily feed intake (ADFI), and feed conversion ratio (FCR). At the end of the feeding trial (80 weeks of age), 6 laying hens per replicate was randomly selected and challenged with diquat. To validate the successful establishment of the oxidative stress model, laying performance was further monitored daily for 7 consecutive days after diquat injection. The daily laying rate during this post-challenge period was calculated.

### Serum intestinal permeability markers

Serum levels of diamine oxidase (DAO), D-lactate (DLA), and lipopolysaccharide (LPS) were measured to evaluate gut barrier integrity and endotoxemia. DAO and DLA were quantified using ELISA kits (Jiancheng Bioengineering Institute, Nanjing, China) following the manufacturer’s protocols. The level of LPS was measured using a chromogenic limulus amebocyte lysate assay kit (Xiamen Bioendo Technology, Xiamen, China).

### Morphological examination

Jejunal segments were paraffin-embedded, sectioned at 5 μm, and stained with hematoxylin and eosin (HE) to assess villus height (tip to crypt base), crypt depth (crypt base to submucosa), and villus-to-crypt ratio. Images were captured using an Olympus microscope (Tokyo, Japan), and 10 well-oriented villi per section were measured with ImageJ software, as previously described [38]. Periodic acid-Schiff (PAS) staining was used to quantify mucus layer thickness. For intestinal organoids, well-developed organoids (d 5–6 after passage) were observed and photographed under an inverted light microscope (Olympus, Tokyo, Japan) at 4× magnification. The number of organoids per microscopic field was counted in 6 randomly selected fields per well to quantify organoid formation efficiency. In addition, organoids were collected, fixed in 4% paraformaldehyde, embedded in paraffin, sectioned at 5 μm, and stained with hematoxylin and eosin to evaluate their internal morphology and structural integrity. All images were captured under the same microscope.

### Transmission electron microscopy (TEM) examination

Jejunal samples fixed in 2.5% glutaraldehyde were post-fixed in 1% osmium tetroxide, dehydrated, and embedded in epoxy resin. Ultrathin sections (70 nm) were stained with uranyl acetate and lead citrate and examined using a JEOL-JEM-1200EX transmission electron microscope (Peabody, MA, USA) to evaluate mitochondrial morphology and tight junction structures in enterocytes. 20 mitochondria per group were randomly selected and scored on a scale of 0 (normal) to 4 (severe damage) based on cristae integrity and matrix density. The percentage of normal mitochondria and average mitochondrial area were quantified using ImageJ software.

### Immunohistochemistry

Paraffin-embedded jejunal sections (5 μm) were deparaffinized, rehydrated, and antigen-retrieved in citrate buffer (pH 6.0) by microwave heating. Endogenous peroxidase was blocked with 3% H₂O₂ for 15 min, and non-specific binding was blocked with 5% BSA for 30 min. Sections were incubated overnight at 4 °C with primary antibody against 4-HNE (rabbit polyclonal, 1:200; Abcam, ab46545). For intestinal organoids, organoids were fixed in 4% paraformaldehyde, embedded in paraffin, and sectioned at 5 μm, then processed similarly. Primary antibodies used were anti-ZO-1 (rabbit polyclonal, 1:150; Abcam, ab216880) and anti-PCNA (mouse monoclonal, 1:200; Abcam, ab29). After washing, sections were incubated with HRP-conjugated secondary antibodies (1:500) for 1 h at room temperature. Color was developed with DAB, counterstained with hematoxylin, and imaged at 200× magnification. Negative controls were performed by omitting the primary antibody. The percentage of positive areas was quantified using ImageJ software.

### Immunofluorescence and TUNEL assays

Jejunal tissues were fixed in 4% paraformaldehyde, dehydrated in 30% sucrose, embedded in OCT compound, and cryosectioned at 5–10 μm. Sections were permeabilized with 0.1% Triton X-100, blocked with 5% goat serum, and incubated overnight at 4 °C with primary antibodies, followed by fluorescent secondary antibodies for 1 h at room temperature. For intestinal organoids, well-developed organoids (d 5–6 after passage) were fixed in 4% paraformaldehyde for 30 min, permeabilized with 0.5% Triton X-100 for 30 min, and blocked with 5% goat serum for 1 h, then incubated with primary antibodies overnight at 4 °C and fluorescent secondary antibodies for 2 h, as previously described [39]. TUNEL assays were performed on both tissues and organoids using a TUNEL kit (Roche, Basel, Switzerland) according to the manufacturer’s instructions. All samples were counterstained with DAPI and imaged under a fluorescence microscope (BX-61, Olympus, Center Valley, PA, USA). Fluorescence intensity was quantified in six randomly selected images per group using ImageJ software.

### Gene expression analysis

Total RNA was extracted from jejunal mucosa and intestinal organoids using a FreeZol Reagent kit (Vazyme, China), and cDNA was synthesized with a cDNA synthesis kit (Vazyme, China). Quantitative real-time PCR (qPCR) was performed on a Real-Time PCR System (Applied Biosystems, Carlsbad, CA, USA) using Taq Pro Universal SYBR qPCR Master Mix (Vazyme, China). Relative mRNA expression was calculated using the 2^−ΔΔCt^ method with β-actin as the reference gene after validation of its stable expression across groups [40]. The primer sequences used for qPCR are provided in Table S3.

### Western blotting

Jejunal tissues were lysed in RIPA buffer (Beyotime, P0013B). Protein concentration was determined by BCA assay (Beyotime, P0012). Equal protein amounts (30–50 μg) were separated by SDS-PAGE, transferred to PVDF membranes, and blocked with 5% non-fat milk in TBST for 1 h, as previously described [41]. Membranes were incubated overnight at 4 °C with primary antibodies: anti-Nrf2 (1:1,000; Proteintech, 16396-1-AP), anti-HO-1 (1:1,000; Proteintech, 10701-1-AP), anti-GPX4 (1:1,000; Proteintech, 67763-1-lg), anti-phospho-NF-κB p65 (1:1,000; Thermo Fisher, 600-400-271), anti-IL-10 (1:1,000; Proteintech, 82191-3-RR), anti-BAX (1:1,000; Proteintech, 60267-1-Ig), anti-BCL2 (1:1,000; Proteintech, 26593-1-AP), anti-LGR5 (1:1,000; Proteintech, 30007-1-AP), anti-β-catenin (1:2,000; Proteintech, 17565-1-AP), and anti-β-actin (1:5,000; Proteintech, 66009-1-lg). After TBST washes, HRP-conjugated secondary antibodies were applied for 1 h. Bands were visualized by an imaging system (Tanon, China). Band intensities were quantified with ImageJ and normalized to β-actin.

### Jejunal inflammatory cytokine quantification

Jejunal mucosa was homogenized in PBS, and supernatants were collected after centrifugation at 4,000 r/min for 10 min at 4 °C, as previously described [42]. Levels of IL-1β, TNF-α, IL-6, and IL-10 were measured using commercial ELISA kits (Nanjing Jiancheng Bioengineering Institute, Nanjing, China) according to the manufacturer’s instructions.

### Antioxidant capacity

Jejunal mucosa (0.1 g) was homogenized in 0.9 mL chilled PBS and centrifuged at 4,000 r/min for 10 min at 4 °C. Serum samples were analyzed directly. For intestinal organoids, well-developed organoids (d 5–6 after passage) were collected after treatments, washed with ice-cold PBS, and homogenized in lysis buffer. All supernatants were used for subsequent assays. The levels of malondialdehyde (MDA; A003-1-2), total antioxidant capacity (T-AOC; A015-1-2), and the activities of superoxide dismutase (SOD; A001-1-2) and glutathione peroxidase (GSH-Px; A005-1-2) in jejunal mucosa, serum, and organoids were measured using commercial kits (Nanjing Jiancheng Bioengineering Institute, Nanjing, China), as previously described [43]. Total protein concentration was determined using a BCA protein assay kit (A045-3, Nanjing Jiancheng Bioengineering Institute, Nanjing, China), and all values were normalized to protein content.

### Mitochondrial analyses

Jejunal mitochondria and intestinal organoids mitochondria were isolated using a mitochondrial isolation kit (Beyotime, Shanghai, China). Tissue was homogenized in extraction buffer, centrifuged at 1,000–2,000 × *g* to remove debris, and the supernatant was centrifuged at 10,000–12,000 × *g* to pellet mitochondria. The level of ROS was measured using dichlorohydro-fluorescein diacetate at 485 nm excitation and 530 nm emission, expressed as dichlorofluorescein fluorescence intensity. Mitochondrial membrane potential (MMP) was assessed with a JC-1 assay kit (Beyotime), with MMP expressed as the red/green fluorescence ratio. Mitochondrial DNA (mtDNA) content was quantified via qPCR using primers for mtDNA-specific genes, normalized to nuclear DNA. Total mitochondrial protein concentration was determined using a BCA protein assay. ATP content was measured using an enhanced ATP assay kit based on luciferase luminescence (Beyotime, S0027). ATP content was expressed as nmol/mg protein. Activities of mitochondrial respiratory chain complexes I and IV were measured using commercial assay kits (Solarbio Biotechnology, Beijing, China). Complex I (NADH-CoQ reductase) activity was measured by monitoring the decrease in NADH absorbance at 340 nm. Complex IV (Cytochrome c oxidase) activity was determined by measuring the oxidation of reduced cytochrome c at 550 nm. All assays were performed in triplicate at 30 °C using a microplate reader (BioTek Synergy H1). Enzyme activities were expressed as nmol/min/mg mitochondrial protein.

### 16S rRNA sequencing

DNA was extracted from cecal contents using a QIAamp DNA Stool Mini Kit (Qiagen, Hilden, Germany). The V3–V4 region of the 16S rRNA gene was amplified and sequenced on an Illumina MiSeq platform (San Diego, CA, USA), as previously described [44]. Sequencing depth was rarefied to 50,000 reads per sample. The beta diversity of the microbiota and linear discriminant analysis (LDA) was performed using the QIIME2 and LDA effect size analysis (LEfSe) softwares, respectively. Relative abundance and statistical difference analyses of microbiota at the phylum and genus levels, as well as correlation analyses and heat mapping, were performed by R software.

### Metabolomics analysis

#### Untargeted metabolomics analysis

Cecal contents were analyzed for untargeted metabolomics using liquid chromatography-mass spectrometry (LC-MS). Samples were extracted in 80% methanol, and metabolites were separated on a Waters ACQUITY UPLC HSS T3 column (2.1 mm × 100 mm, 1.8 μm) using a Waters UPLC system coupled to a Q-Exactive Orbitrap mass spectrometer (Thermo Fisher Scientific, Waltham, MA, USA), as previously described [45]. Data were acquired in positive and negative ion modes, raw data were processed with XCMS software for peak detection, alignment, and integration. Differential metabolites were identified using fold change (FC > 1.5) and Student's *t*-test (*P* < 0.05). Pathway enrichment analysis was performed using the KEGG database in MetaboAnalyst 5.0, with *P*-values adjusted for multiple testing using the Benjamini-Hochberg false discovery rate (FDR) method. Pathways with FDR < 0.05 were considered significantly enriched.

#### Targeted metabolomics analysis

Cecal contents and jejunal mucosa were homogenized in ice-cold methanol:acetonitrile:water (2:2:1, v/v/v) containing internal standards. After centrifugation (14,000 × *g*, 15 min, 4 °C), the supernatant was evaporated and reconstituted in 50% methanol. Targeted quantification of tryptophan metabolites was performed using UHPLC-QqQ-MS/MS (Agilent 1290-6470) with a Waters ACQUITY UPLC BEH C18 column. Detection was conducted in positive ESI mode with multiple reaction monitoring (MRM). Quantification was achieved using external standard curves and internal standard normalization. Results were expressed as nmol/g wet weight.

### Intestinal organoid culture

Intestinal crypts were isolated from the jejunum of 70-week-old laying hens. Briefly, jejunal tissues were washed with ice-cold PBS containing 1% penicillin-streptomycin, minced into small pieces, and incubated in 2 mmol/L EDTA in PBS at 4 °C for 35 min with gentle shaking. Crypts were released by vigorous pipetting, filtered through a 70-μm cell strainer, and centrifuged at 300 × *g* for 5 min. The crypt pellet was resuspended in Matrigel and seeded in 24-well plates. Organoids were cultured in advanced DMEM/F12 supplemented with 10% Noggin-conditioned medium, 10% R-spondin1-conditioned medium, 50 ng/mL EGF, 100 ng/mL Wnt3a, 1× B27, 1× N2, 1 mmol/L N-acetylcysteine, 10 mmol/L HEPES, and 1% penicillin-streptomycin. The culture medium was changed every 2 d, and organoids were passaged every 5–7 d by mechanical dissociation. Organoids were maintained at 37 °C in a humidified incubator with 5% CO_2_.

### Diquat-induced oxidative stress model and interventions in organoids

Well-developed organoids (d 5–6 after passage) were used for experiments. For the diquat (DQ) model, organoids were treated with 150 μmol/L diquat dibromide monohydrate for 24 h to induce oxidative stress. For the * L. reuteri* supernatant intervention (LRSD), *L. reuteri* was cultured in MRS broth to stationary phase, the supernatant was collected, centrifuged at 8,000 × *g* for 10 min, filtered through a 0.22-μm filter, and neutralized to pH 7.0. Organoids were pretreated with 15% (v/v) *L. reuteri* supernatant for 24 h prior to diquat exposure. For the indole-3-acetic acid intervention (IAAD), organoids were pretreated with 20 μmol/L indole-3-acetic acid for 12 h before diquat challenge. Control organoids were treated with equal volumes of vehicle. After treatments, organoids were collected for subsequent analyses.

### Statistical analysis

Data were analyzed using SPSS 26.0 (IBM, Armonk, NY, USA) and expressed as mean ± standard deviation (SD). Data were compared using one-way ANOVA with Tukey’s post-hoc test. Graphs were generated using GraphPad Prism 8.0 (GraphPad Software, San Diego, CA, USA). Significance was set at *P* < 0.05.

## Results

### *L. reuteri* improves production performance and enhances intestinal barrier integrity in diquat-challenged laying hens

During the 10-week feeding trial (71–80 weeks of age), supplementation with *L. reuteri* Y067 significantly improved laying rate, average daily egg mass, and feed conversion ratio in aged laying hens compared with the control group (*P* < 0.05; Table S1). At the end of the feeding trial, laying hens were challenged with diquat to induce oxidative stress. During the subsequent 7-day post-challenge period, diquat administration significantly decreased the laying rate in the DQ group compared with the CON group (*P* < 0.01). Notably, *L. reuteri* pretreatment effectively alleviated this decline (*P* < 0.05; Table S2).

**Fig. 1:**
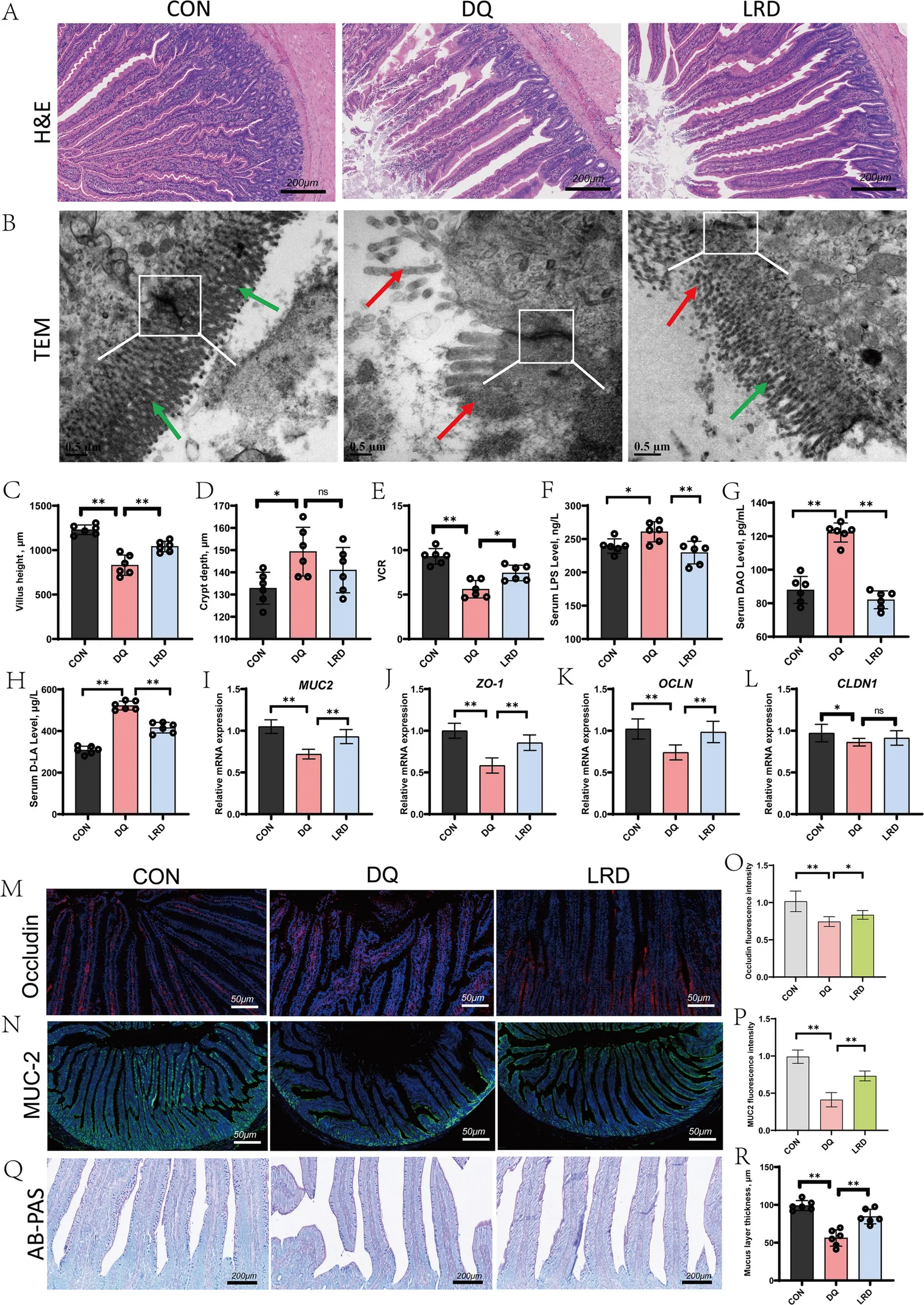
*Limosilactobacillus reuteri* improves production performance and enhances intestinal structure and barrier function in diquat-challenged laying hens. **A** Representative HE-stained jejunal sections. **B** Representative TEM images of jejunal epithelial tight junctions and microvilli. **C**–**E** Quantitative analysis of villus height (**C**), crypt depth (**D**), and villus height/crypt depth ratio (**E**). **F**–**H** Serum levels of LPS, DAO and D-LA. **I**–**L** Relative mRNA expression of barrier-related genes *MUC2* (**I**), *ZO-1* (**J**), *OCLN* (**K**), and *CLDN1* (**L**) in jejunal tissues. **M** and **N** Representative immunofluorescence staining of Occludin and MUC2 in jejunal sections. **O** and **P** Quantitative analysis of Occludin (**O**) and MUC2 (**P**) immunofluorescence intensity. **Q** Representative AB-PAS staining of jejunal mucus layer. **R** Quantitative analysis of mucus layer thickness. Data are presented as mean ± SD (*n* = 6). ^**^P < 0.01, ^*^P < 0.05 vs. DQ group; ns, no significance

Histological examination of jejunal sections stained with HE revealed severe mucosal damage in the DQ group (Fig. 1A). In contrast, the LRD group exhibited improved villus architecture with enhanced height and reduced structural disruption, approaching the intact morphology of the CON group (Fig. 1A). TEM further confirmed barrier ultrastructural impairment in the DQ group (Fig. 1B). *L. reuteri* pretreatment significantly alleviated these changes, evidenced by the increased tight junction structures and preserved microvilli morphology in the LRD group. Morphometric analysis showed that *L. reuteri* supplementation significantly improved villus height and the villus/crypt ratio (*P* < 0.05), although crypt depth remained comparable between the DQ and LRD groups (*P* > 0.05; Fig. 1C–E). Serum permeability markers including LPS, DAO, and D-LA were significantly elevated in the DQ group (*P* < 0.05; Fig. 1F–H). *L. reuteri* pretreatment markedly mitigated these indicators (*P* < 0.01). qPCR analysis demonstrated significant downregulation of barrier-related mRNA expression in the DQ group, including *MUC2*, *ZO-1*, *OCLN*, and *CLDN1* (*P* < 0.05; Fig. 1I–L). *L. reuteri* intervention significantly upregulated the mRNA expression of these genes *(P* < 0.05; Fig. 1I–K). Immunofluorescence staining showed reduced fluorescence intensity of Occludin and MUC2 in the DQ group (Fig. 1M and N). *L. reuteri* pretreatment enhanced fluorescence intensity for both proteins (Fig. 1M and N). Quantitative analysis confirmed significantly lower Occludin and MUC2 fluorescence intensity in the DQ group compared to CON (*P* < 0.01), while *L. reuteri* supplementation improved these levels (*P* < 0.05; Fig. 1O and P). AB-PAS staining revealed a thinned and disrupted mucus layer in the DQ group (Fig. 1Q). *L. reuteri* pretreatment significantly increased mucus layer thickness (*P* < 0.01), approaching CON levels (Fig. 1R).

### *L. reuteri* reshapes the gut microbiota composition in diquat-challenged hens

**Fig. 2:**
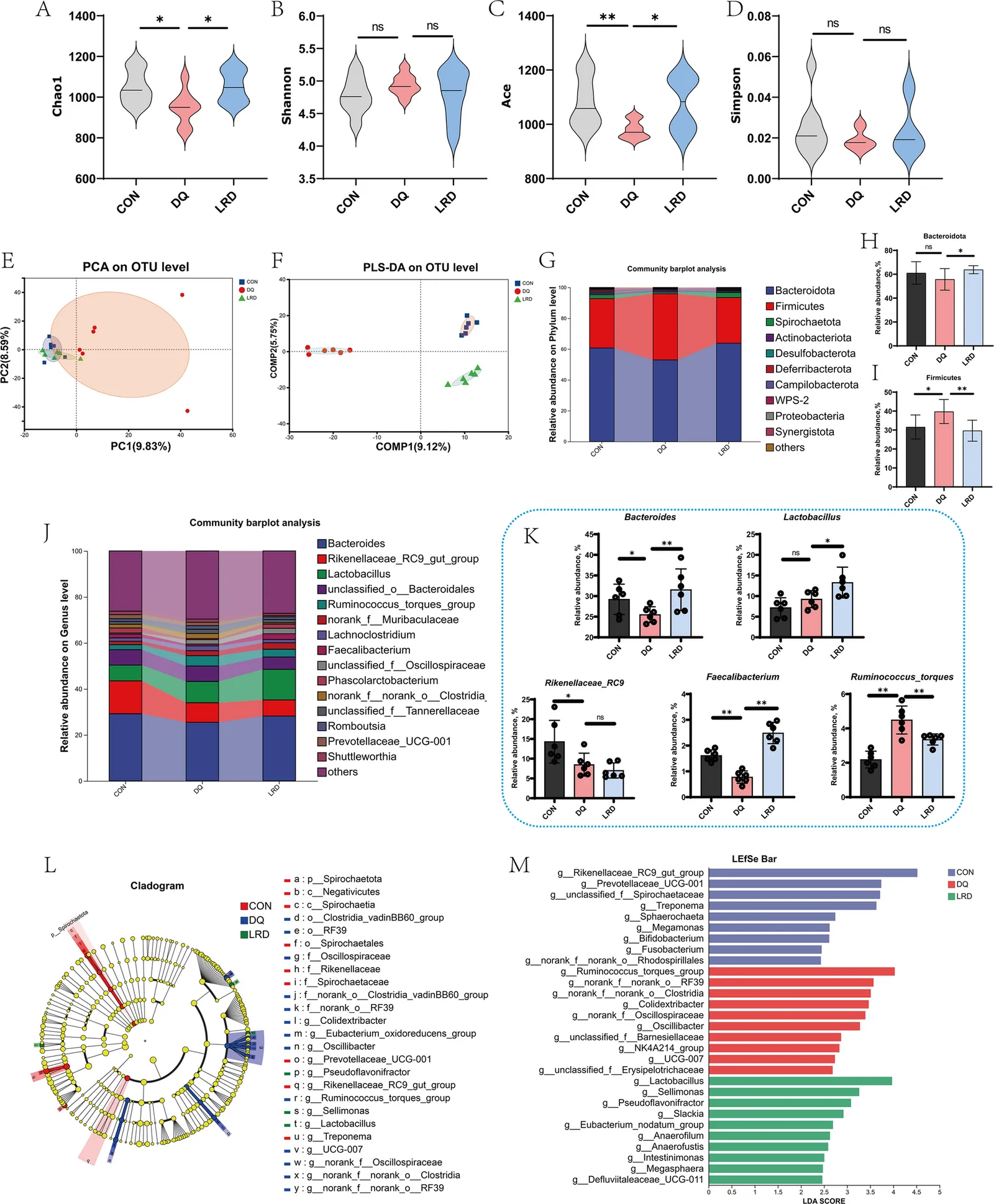
*Limosilactobacillus reuteri* reshapes the gut microbiota composition in diquat-challenged laying hens. **A**–**D** Alpha diversity indices of cecal microbiota. **E** PCoA at the OTU level. **F** PLS-DA plot at the OTU level. **G** Stacked bar plot of microbial composition at the phylum level (top 10 phyla). **H** and **I** Relative abundance of Bacteroidota (**H**) and Firmicutes (**I**) at the phylum level. **J** Stacked bar plot of microbial composition at the genus level (top 15 genera). **K** Relative abundance of key genera. **L** Cladogram showing taxonomic biomarkers. **M** LEfSe score bar plot (LDA > 3.5). Data are presented as mean ± SD (*n* = 6). ^**^P < 0.01, ^*^P < 0.05 vs. DQ group; ns, no significance

Alpha diversity analysis of cecal microbiota revealed significant alterations among groups. The ACE and Chao1 indices, reflecting community richness, were markedly decreased in the DQ group compared to CON group (*P* < 0.05), while the Shannon and Simpson indices showed no significant differences (Fig. 2A–D). *L. reuteri* pretreatment significantly improved ACE and Chao1 indices to levels comparable to the CON group (*P* < 0.05; Fig. 2A and B). Beta diversity analysis demonstrated distinct microbial community structures. Principal coordinate analysis (PCoA) and partial least squares discriminant analysis (PLS-DA) clearly separated the DQ group from the CON and LRD groups, with the LRD group clustering closer to the CON group (Fig. 2E and F). PERMANOVA based on Bray-Curtis dissimilarity further confirmed significant differences in overall microbiota composition among the three groups (*R*^2^ = 0.320, *P* = 0.036).

As for microbiota composition, at the phylum level, diquat challenge increased the relative abundance of Firmicutes and decreased Bacteroidota compared to the CON group (Fig. 2G). Quantitative analysis confirmed a significant elevation in Firmicutes (*P* < 0.05) and reduction in Bacteroidota in the DQ group, with *L. reuteri* pretreatment promoting Bacteroidota abundance and normalizing the Firmicutes/Bacteroidota ratio (*P* < 0.05; Fig. 2H and I). At the genus level, the most abundant genera included *Bacteroides*, *Rikenellaceae_RC9_gut_group*, *Faecalibacterium*, *Lactobacillus*, and *Ruminococcus_torques_group* (Fig. 2J). Quantitative comparison showed that diquat exposure significantly decreased *Bacteroides, Faecalibacterium,* and *Rikenellaceae_RC9_gut_group* while increasing *Ruminococcus_torques_group* (*P* < 0.05; Fig. 2K). Notably, *Lactobacillus* and *Faecalibacterium* abundance increased in the LRD group (*P* < 0.01; Fig. 2K). Linear discriminant analysis effect size (LEfSe) identified taxonomic biomarkers across groups. The cladogram illustrated enrichment of Spirochaetota phylum and beneficial genera such as Rikenellaceae and Spirochaetales in the CON group, *Ruminococcus_torques_group* and *Colidextribacter* in the DQ group, and *Lactobacillus* in the LRD group (Fig. 2L). LDA score analysis (LDA > 3.5) confirmed *Rikenellaceae_RC9_gut_group* as the primary biomarker in the CON group, *Ruminococcus_torques_group* in the DQ group, and *Lactobacillus* in the LRD group (Fig. 2M). These findings demonstrate that *L. reuteri* pretreatment effectively mitigates diquat-induced gut dysbiosis by improving microbial diversity and enriching beneficial taxa, particularly *Lactobacillus*.

### *L. reuteri* remodels the intestinal metabolome with significant enrichment in tryptophan metabolism

**Fig. 3:**
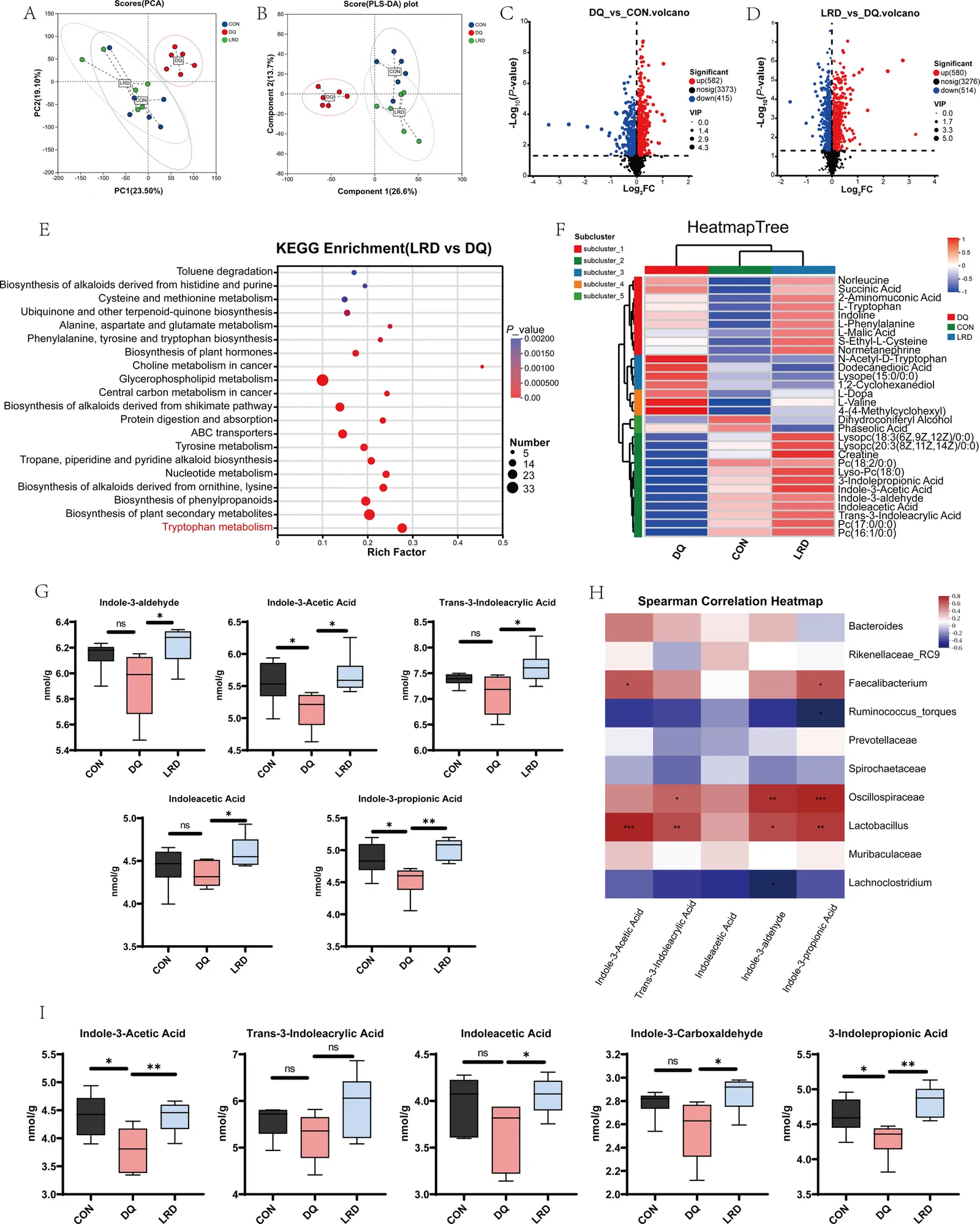
*Limosilactobacillus reuteri* remodels the intestinal metabolome with significant enrichment in tryptophan metabolism. **A** PCA score plot of cecal metabolome profiles. **B** OPLS-DA score plot of cecal metabolome profiles. **C** Volcano plot of differential metabolites in DQ vs. CON comparison (*P* < 0.05). **D** Volcano plot of differential metabolites in LRD vs. DQ comparison (*P* < 0.05). **E** KEGG pathway enrichment analysis bubble plot showing significantly enriched pathways. **F** Hierarchical clustering heatmap of the top 30 differential metabolites across groups. **G** Targeted metabolomics analysis of representative tryptophan metabolites contents on the cecum. **H** Spearman correlation heatmap between the top 10 abundant bacterial genera and five key tryptophan metabolites in the cecal contents. **I** Targeted metabolomics analysis of representative tryptophan metabolites contents on the jejunum. Data are presented as mean ± SD (*n* = 6). ^**^P < 0.01, ^*^P < 0.05 vs. DQ group

Non-targeted metabolomics analysis of cecal contents revealed distinct metabolic profiles among the three groups. Principal component analysis (PCA) showed clear separation between the DQ group and the CON group, while the LRD group clustered closer to the CON group (Fig. 3A). Orthogonal partial least squares discriminant analysis (OPLS-DA) further confirmed significant differences in metabolic patterns, with good model fit and predictability (Fig. 3B). Volcano plot analysis (*P* < 0.05) identified a total of 582 upregulated and 415 downregulated metabolites in the DQ vs. CON comparison, and 580 upregulated and 129 downregulated metabolites in the LRD vs. DQ comparison (Fig. 3C and D). These results indicate that diquat induced widespread metabolic disturbances, whereas *L. reuteri* pretreatment partially normalized these alterations. KEGG pathway enrichment analysis revealed that tryptophan metabolism was significantly enriched in the LRD group compared to the DQ group (FDR-adjusted *P* = 1.314 × 10^−^^9^; Fig. 3E). This pathway showed the highest enrichment score and was strongly associated with tryptophan remodeling under oxidative stress. Hierarchical clustering heatmap of the top 30 differential metabolites demonstrated clear group separation, with tryptophan metabolism-related metabolites markedly upregulated in the LRD group (Fig. 3F). Cecum-targeted metabolomics confirmed significant increases in key tryptophan metabolites, including IAA, indole-3-aldehyde, and IPA in the LRD group compared to the DQ group (*P* < 0.05; Fig. 3G). Spearman correlation analysis further revealed that *Lactobacillus* was positively correlated with these metabolites (especially IAA and IPA), whereas *Ruminococcus_torques_group* showed negative correlations (*P* < 0.05; Fig. 3H). Beneficial genera such as *Faecalibacterium* and *Oscillospiraceae* also displayed strong positive correlations with several tryptophan metabolites. To further validate the changes in tryptophan metabolites at the site of jejunum, we performed targeted metabolomics on jejunal mucosa. As shown in Fig. 3I, the concentrations of IAA and IPA were also significantly increased in the jejunum of the LRD group compared with the DQ group (*P* < 0.01).

### *L. reuteri* alleviates diquat-induced mitochondrial dysfunction, impaired biogenesis, dysregulated dynamics, and mitophagy imbalance

**Fig. 4:**
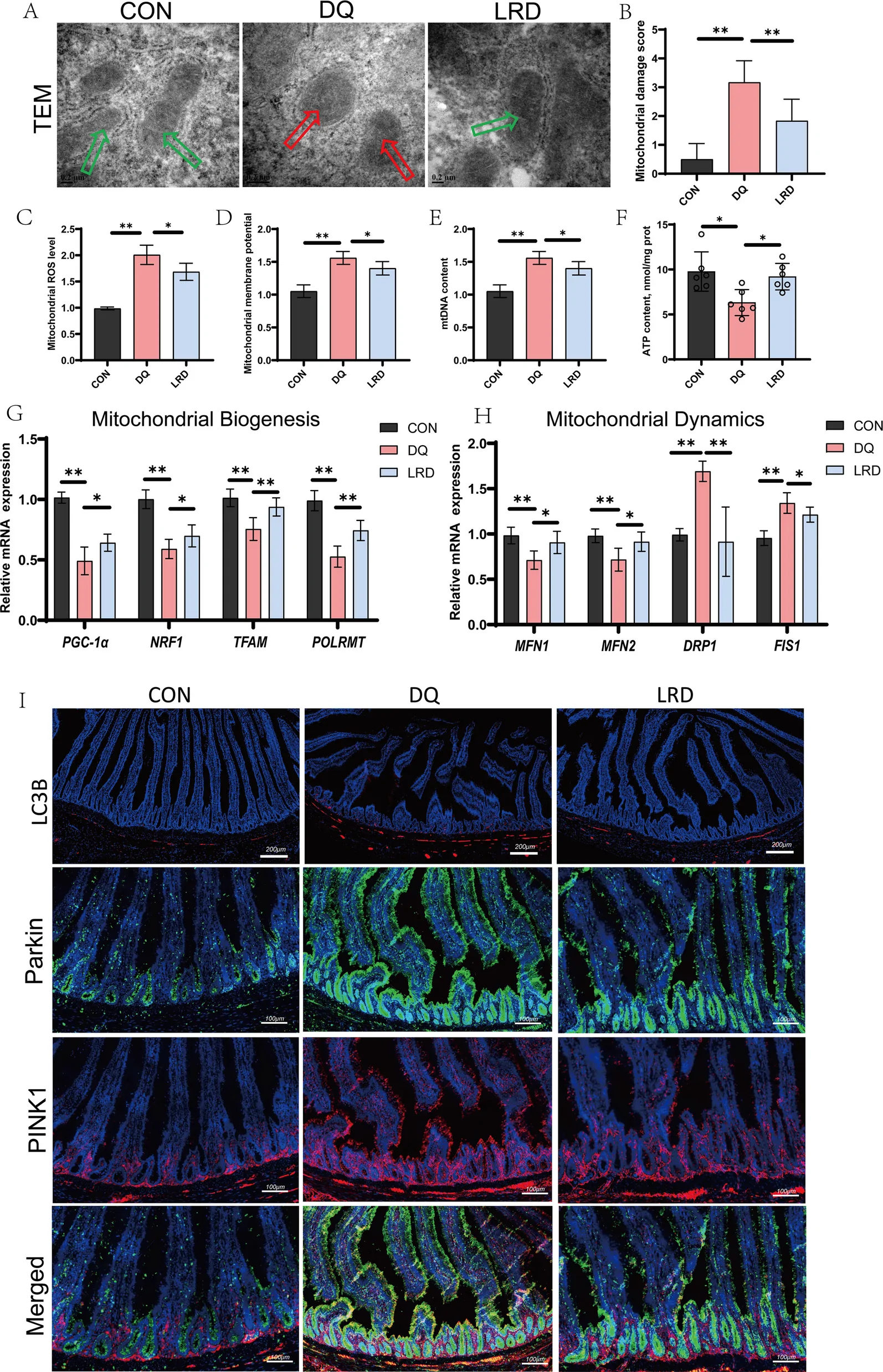
*Limosilactobacillus reuteri* alleviates mitochondrial dysfunction, impaired biogenesis, dysregulated dynamics, and mitophagy alterations in diquat-challenged laying hens. **A** Representative TEM images of mitochondrial ultrastructure in jejunal epithelial cells. **B** Mitochondrial damage score. **C**–**F** Quantitative analysis of mitochondrial ROS level (**C**), mitochondrial membrane potential (MMP; **D**), mtDNA copy number (**E**), and ATP content (**F**). **G** Relative mRNA expression of mitochondrial biogenesis-related genes *PGC-1α*,* NRF1*,* TFAM*, and *POLRMT*. **H** Relative mRNA expression of mitochondrial dynamics-related genes *MFN1*,* MFN2*, *DRP1*, and *FIS1*. **I** Representative immunofluorescence staining of mitophagy markers LC3B, Parkin, PINK1, and merged images in jejunal sections. Data are presented as mean ± SD (*n* = 6). ^**^P < 0.01, ^*^P < 0.05 vs. DQ group

TEM revealed severe mitochondrial ultrastructural damage in the DQ group. In contrast, mitochondria in the CON and LRD groups exhibited intact morphology with well-organized cristae and normal matrix density (Fig. 4A). The mitochondrial damage score was markedly higher (*P* < 0.01) in the DQ group and was significantly reduced in the LRD group (Fig. 7B). Diquat challenge significantly increased mitochondrial ROS production, and decreased mitochondrial membrane potential, mtDNA content, and ATP production (*P* < 0.05) compared to the control group (Fig. 4C–F). *L. reuteri* pretreatment markedly attenuated these changes, reducing ROS, restoring MMP, increasing mtDNA copy number, and elevating ATP levels (*P* < 0.05) relative to the DQ group (Fig. 4C–F). qPCR analysis demonstrated that diquat exposure downregulated mitochondrial biogenesis-related mRNA expression, including *PGC-1α*, *NRF1*, *TFAM*, and *POLRMT* (*P* < 0.05; Fig. 4G). *L. reuteri* supplementation significantly upregulated these genes (*P* < 0.05; Fig. 4G), indicating enhanced mitochondrial biogenesis. Regarding mitochondrial dynamics, diquat increased fission genes *DRP1* and *FIS1* (*P* < 0.01) while decreasing fusion genes *MFN1* and *MFN2* (*P* < 0.05; Fig. 4H). *L. reuteri* ameliorated this imbalance by downregulating *DRP1* and *FIS1* (*P* < 0.05) and upregulating *MFN1* and *MFN2* (*P* < 0.05; Fig. 4H). Furthermore, immunofluorescence staining showed that diquat increased the expression of mitophagy markers LC3B, Parkin, and PINK1 in the jejunal epithelium (Fig. 4I). *L. reuteri* supplementation effectively attenuated the excessive mitophagy.

### *L. reuteri* enhances antioxidant capacity and activates the Nrf2 pathway in diquat-challenged laying hens

**Fig. 5:**
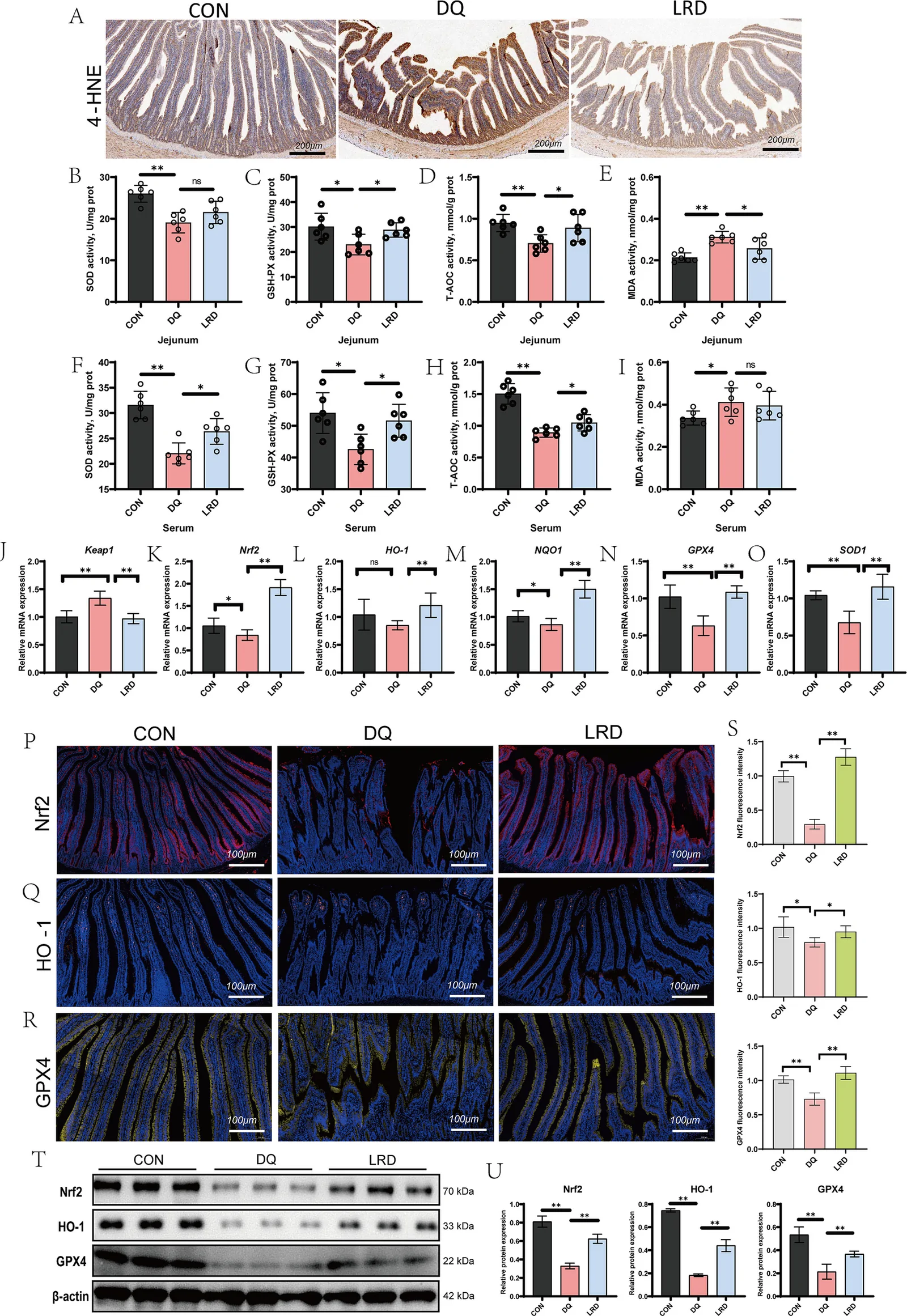
*Limosilactobacillus reuteri* enhances antioxidant capacity and activates the Nrf2 pathway in diquat-challenged laying hens. **A** Representative immunohistochemical staining of 4-HNE. **B**–**E** Jejunal antioxidant parameters: SOD (**B**), GSH-Px (**C**), T-AOC (**D**), and MDA (**E**). **F**–**I** Serum antioxidant parameters: SOD (**F**), GSH-Px (**G**), T-AOC (**H**), and MDA (**I**). **J**–**O** Relative mRNA expression of Nrf2 pathway-related genes: *Keap1*, *Nrf2*, *HO-1*, *NQO1*, *GPX4*, and *SOD1*. **P**–**R** Representative immunofluorescence staining of Nrf2, HO-1, GPX4 in jejunal sections. **S** Quantitative analysis of Nrf2, HO-1, and GPX4 immunofluorescence intensity. **T** and **U** Representative Western blot bands and quantitative densitometry of Nrf2, HO-1, and GPX4 proteins normalized to β-actin. Data are presented as mean ± SD (*n* = 6). ^**^P < 0.01, ^*^P < 0.05 vs. DQ group; ns, no significance

Immunohistochemical staining for 4-HNE showed intense brown staining in the villi and crypts of the DQ group. In the LRD group, 4-HNE staining intensity was markedly reduced, with improved villus morphology approaching the CON group (Fig. 5A). In jejunal tissues, diquat challenge significantly decreased SOD, GSH-Px, and T-AOC activities, while increasing MDA content compared to the CON group. *L. reuteri* pretreatment significantly ameliorated GSH-Px and T-AOC activities (*P* < 0.05) and reduced MDA levels (*P* < 0.05). Serum antioxidant parameters followed a similar pattern: diquat exposure reduced SOD, GSH-Px, and T-AOC activities while elevating MDA content. *L. reuteri* intervention significantly improved SOD, T-AOC and GSH-Px activities (*P* < 0.05; Fig. 5F–I).

qPCR analysis of Nrf2 pathway-related genes revealed that diquat downregulated *Nrf2*, *NQO1*, *GPX4*, and *SOD1* mRNA expression. *L. reuteri* pretreatment significantly upregulated *Nrf2*, *HO-1*, and *NQO1*, with the restoration of *GPX4* and *SOD1* (*P* < 0.05; Fig. 5J–O). Immunofluorescence staining demonstrated reduced nuclear translocation of Nrf2 and cytoplasmic expression of HO-1 and GPX4 in the DQ group. *L. reuteri* pretreatment enhanced Nrf2 nuclear localization and increased HO-1 and GPX4 fluorescence intensity (Fig. 5P–R). Quantitative analysis confirmed significantly lower Nrf2, HO-1, and GPX4 fluorescence intensity in the DQ group compared to CON (*P* < 0.05), with *L. reuteri* improving these levels (*P* < 0.05; Fig. 5S). Western blot analysis revealed that diquat downregulated Nrf2, HO-1, and GPX4 protein expression. *L. reuteri* pretreatment significantly upregulated these proteins (*P* < 0.01 vs. DQ; Fig. 5T and U), with Nrf2 and HO-1 levels restored to near CON values and GPX4 partially recovered.

### *L. reuteri* suppresses diquat-induced inflammatory response and NF-κB signaling

**Fig. 6:**
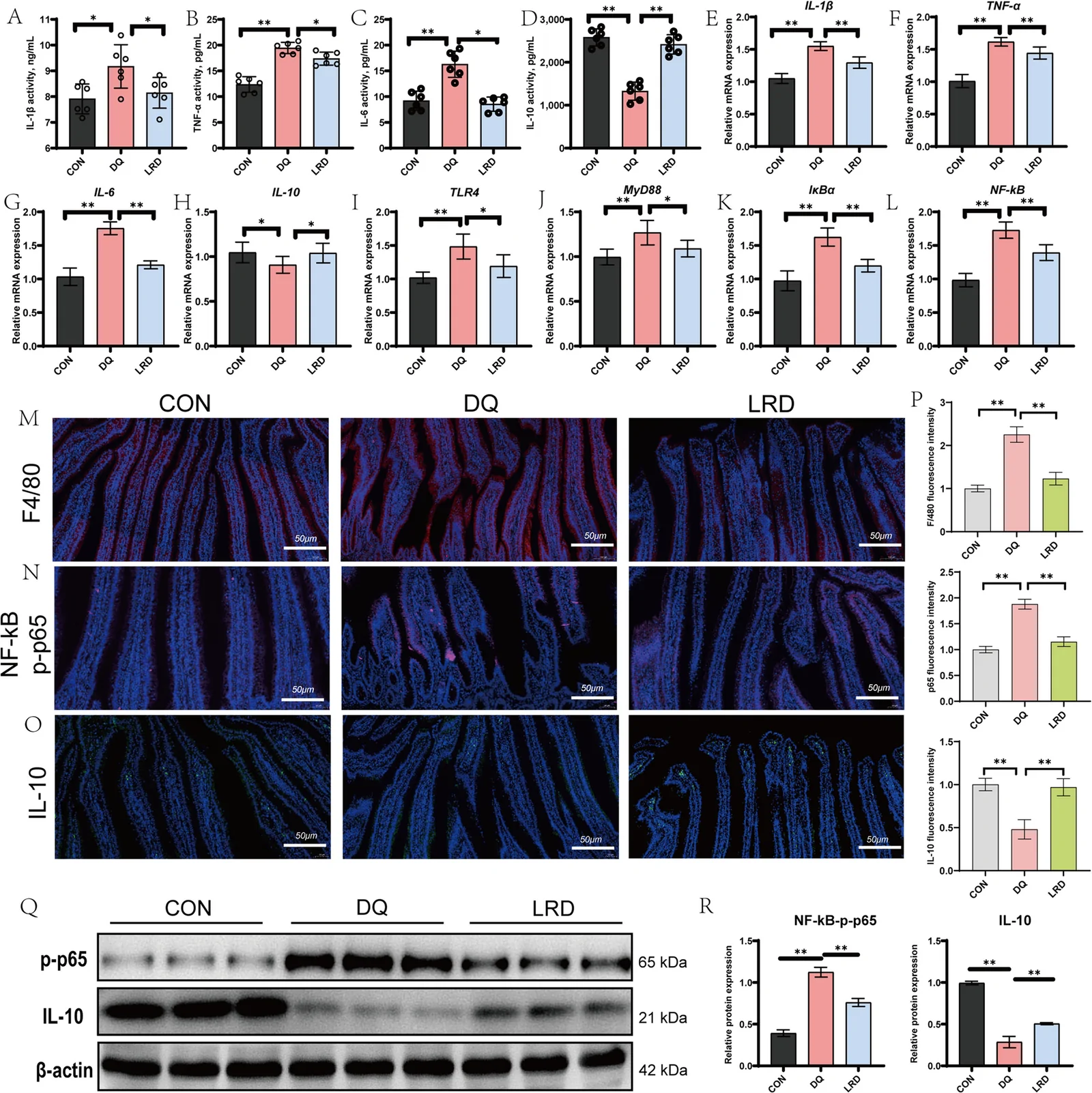
*Limosilactobacillus reuteri* suppresses diquat-induced inflammatory response and NF-κB signaling activation. **A**–**D** Jejunal protein levels of pro-inflammatory cytokines IL-1β, TNF-α, IL-6, and anti-inflammatory cytokine IL-10. **E**–**H** Relative mRNA expression of *IL-1β*, *TNF-α*, *IL-6*, and *IL-10*. **I**–**L** Relative mRNA expression of NF-κB pathway-related genes *TLR4*, *MYD88*, *IκBα*, and *NF-κB*. **M**–**O** Representative immunofluorescence staining of macrophage marker F4/80, phosphorylated p65 and IL-10. **P** Quantitative analysis of F4/80, p-p65 NF-κB, and IL-10 immunofluorescence intensity. **Q** Representative Western blot bands of p-p65 NF-κB and IL-10 proteins normalized to β-actin. **R** Quantitative densitometry of p-p65 NF-κB and IL-10 protein expression. Data are presented as mean ± SD (*n* = 6). ^**^P < 0.01, ^*^P < 0.05 vs. DQ group; ns, no significance

ELISA of jejunal tissues showed that DQ challenge significantly increased the protein levels of pro-inflammatory cytokines IL-1β, TNF-α, and IL-6 (*P* < 0.05), while decreasing the anti-inflammatory cytokine IL-10 compared to the CON group (*P* < 0.01; Fig. 6A–D). LRD pretreatment markedly reduced IL-1β, TNF-α, and IL-6 levels (*P* < 0.05) and elevated IL-10 content (*P* < 0.01) relative to the CON group (Fig. 6A–D). Consistent with protein data, qPCR analysis revealed that *L. reuteri* pretreatment significantly downregulated *IL-1β*, *TNF-α*, and *IL-6* mRNA expression (*P* < 0.05) and upregulated *IL-10* mRNA expression (*P* < 0.05; Fig. 6E–H). Further qPCR analysis of the TLR4/NF-κB pathway showed that diquat significantly upregulated *TLR4*, *MYD88*, *IκBα*, and *NF-κB* mRNA expression. *L. reuteri* intervention significantly reduced *TLR*4, *MYD88*, *IκBα*, and *NF-κB* mRNA levels (*P* < 0.05; Fig. 6I–L).

Immunofluorescence staining demonstrated increased macrophage infiltration (F4/80-positive cells) and nuclear translocation of phosphorylated p65 (p-p65 NF-κB) in the DQ group, with reduced IL-10 expression. *L. reuteri* pretreatment decreased F4/80 and p-p65 fluorescence intensity while enhancing IL-10 staining (Fig. 6M–O). Quantitative analysis confirmed significantly higher F4/80 and p-p65 intensity and lower IL-10 intensity in the DQ group compared to CON (*P* < 0.01), with *L. reuteri* alleviating these levels (*P* < 0.05; Fig. 6P). Western blot analysis showed increased phosphorylated p65 (p-p65 NF-κB) and decreased IL-10 protein expression in the DQ group (*P* < 0.01 vs. CON; Fig. 6Q and R). *L. reuteri* pretreatment significantly reduced p-p65 and restored IL-10 protein levels (*P* < 0.01 vs. DQ; Fig. 6Q and R).

### *L. reuteri* inhibits apoptosis and promotes proliferation in diquat-challenged laying hens

**Fig. 7:**
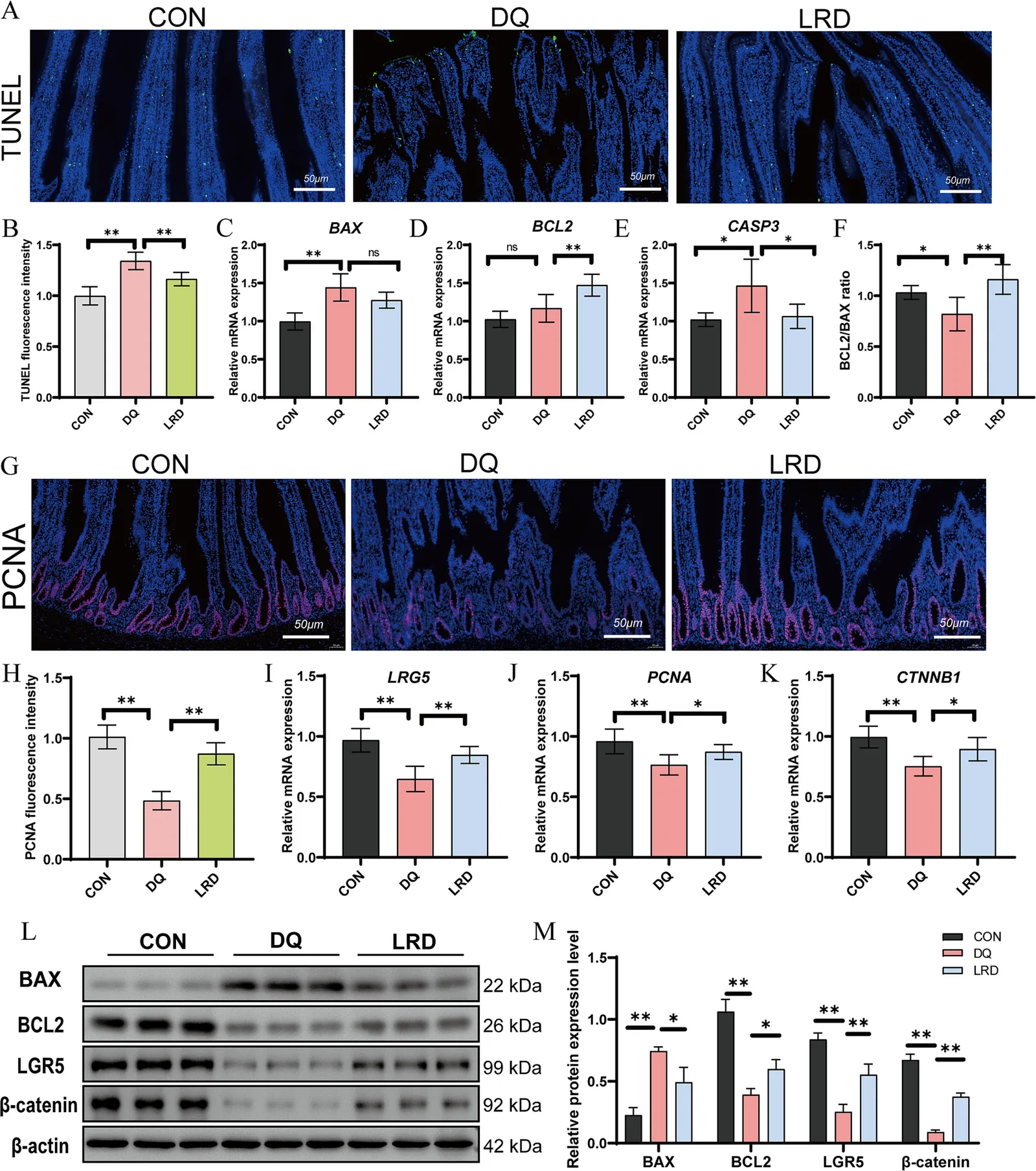
*Limosilactobacillus reuteri* inhibits apoptosis and promotes proliferation in diquat-challenged laying hens. **A** Representative TUNEL immunofluorescence staining. **B** Quantitative analysis of TUNEL fluorescence intensity. **C**–**E** Relative mRNA expression of apoptosis-related genes *BAX*, *BCL2*, and *CASP3*. **F** BCL2/BAX ratio. **G** Representative immunofluorescence staining of PCNA. **H** Quantitative analysis of PCNA fluorescence intensity. **I**–**K** Relative mRNA expression of proliferation-related genes *LGR5*, *PCNA*, and *CTNNB1*. **L** Representative Western blot bands of BAX, BCL2, LGR5, and β-catenin proteins. **M** Quantification of protein expression levels normalized to β-actin. Data are presented as mean ± SD (*n* = 6). ^**^P < 0.01, ^*^P < 0.05 vs. DQ group; ns, no significance

TUNEL staining revealed a significant increase in apoptotic cells in the DQ group. LRD pretreatment markedly reduced TUNEL-positive cells, with fluorescence intensity approaching the CON group (Fig. 7A and B). qPCR analysis of apoptosis-related genes showed that diquat significantly upregulated *BAX* and *CASP3* mRNA expression (*P* < 0.05; Fig. 7C–E). The BCL2/BAX ratio was markedly decreased in the DQ group (*P* < 0.05; Fig. 7F). *L. reuteri* pretreatment significantly downregulated *CASP3* mRNA expression, upregulated *BCL2* mRNA expression (*P* < 0.01), and restored the BCL2/BAX ratio (*P* < 0.01; Fig. 7C–F). Regarding cell proliferation, diquat exposure significantly decreased PCNA fluorescence intensity (Fig. 7G and H). qPCR analysis of proliferation-related genes revealed that diquat significantly downregulated *LGR5*, *PCNA*, and *CTNNB1 *(β-catenin) mRNA expression (*P* < 0.01). *L. reuteri* pretreatment significantly upregulated the mRNA expression of these genes (*P* < 0.05; Fig. 7I–K). Western blot analysis further confirmed these findings at the protein level (Fig. 7L and M). Diquat significantly increased BAX protein expression while decreasing BCL2, LGR5, and β-catenin protein levels. Supplementation with *L. reuteri* markedly downregulated BAX and upregulated BCL2, LGR5, and β-catenin protein expression (*P* < 0.05).

### Mantel test and Procrustes analysis show associations between gut microbiota, tryptophan metabolites, mitochondrial function, and intestinal barrier following *L. reuteri* supplementation

**Fig. 8:**
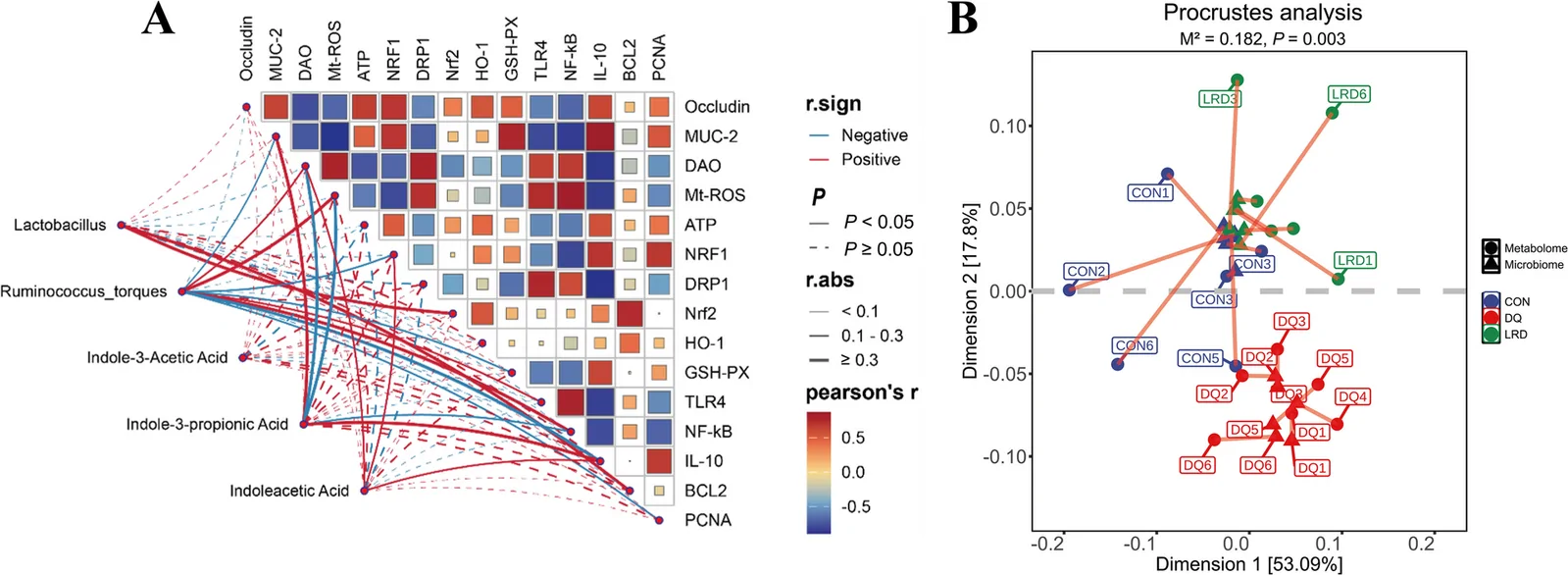
Mantel test and Procrustes analysis show associations between gut microbiota, tryptophan metabolites, mitochondrial function, and intestinal barrier following *Limosilactobacillus reuteri* supplementation. **A** Correlation network and heatmap integrating gut microbiota, tryptophan metabolites, and host phenotypic indicators. Only significant correlations (*|r*| > 0.3, *P* < 0.05) are displayed. Red lines represent positive correlations, blue dashed lines represent negative correlations, and line thickness is proportional to the absolute value of the correlation coefficient. **B** Procrustes analysis comparing gut microbiome (triangles) and metabolome (circles) ordinations across CON (blue), DQ (red), and LRD (green; *L. reuteri*-supplemented) groups. Connecting lines link paired microbiome and metabolome data points from the same biological sample; shorter lines indicate greater concordance between datasets. M^2^ and *P*-values from the Procrustes test are shown above the plot

To further elucidate the relationships among microbiota composition, tryptophan metabolites, and downstream intestinal parameters, we performed Mantel test correlation analysis combined with a Pearson correlation heatmap (Fig. 8A), as well as Procrustes analysis (Fig. 8B). As shown in Fig. 8A, Mantel test analysis showed that *Lactobacillus* exhibited strong positive correlations with multiple protective indicators, including barrier proteins (MUC2 and Occludin), mitochondrial ATP content, biogenesis factor NRF1, antioxidant markers (Nrf2, HO-1, and GSH-Px), anti-inflammatory cytokine IL-10, and proliferation marker PCNA. In contrast, *Ruminococcus_torques_group* showed significant negative correlations with these parameters and positive correlations with oxidative stress and inflammatory markers (Mt-ROS, NF-κB, IL-1β). Among tryptophan metabolites, indole-3-acetic acid, indole-3-propionic acid, and indoleacetic acid displayed strong positive associations with mitochondrial function (ATP, NRF1), antioxidant capacity (HO-1, GSH-Px), and barrier integrity (MUC2, Occludin), while being negatively correlated with ROS, NF-κB signaling.

To further validate the overall concordance between the gut microbiome and metabolome datasets, we performed Procrustes analysis (Fig. 8B). The analysis revealed a significant correlation between microbiome and metabolome ordinations (M^2^ = 0.182, *P* = 0.003), indicating substantial congruence between microbial community structure and the global metabolite profile. Samples from the CON and LRD groups clustered closely together near the plot origin with short connecting vectors between paired microbiome and metabolome points, indicating consistent and stable microbiome-metabolome relationships in these groups. In contrast, DQ group samples were clearly separated along Dimension 1, forming a distinct cluster with longer, more divergent connecting vectors, reflecting a coordinated DQ-induced shift in both microbial composition and metabolite profile away from the CON/LRD configuration.

### Tryptophan metabolites improve mitochondrial function and alleviate oxidative stress, inflammation, and apoptosis in DQ-injured intestinal organoids

**Fig. 9:**
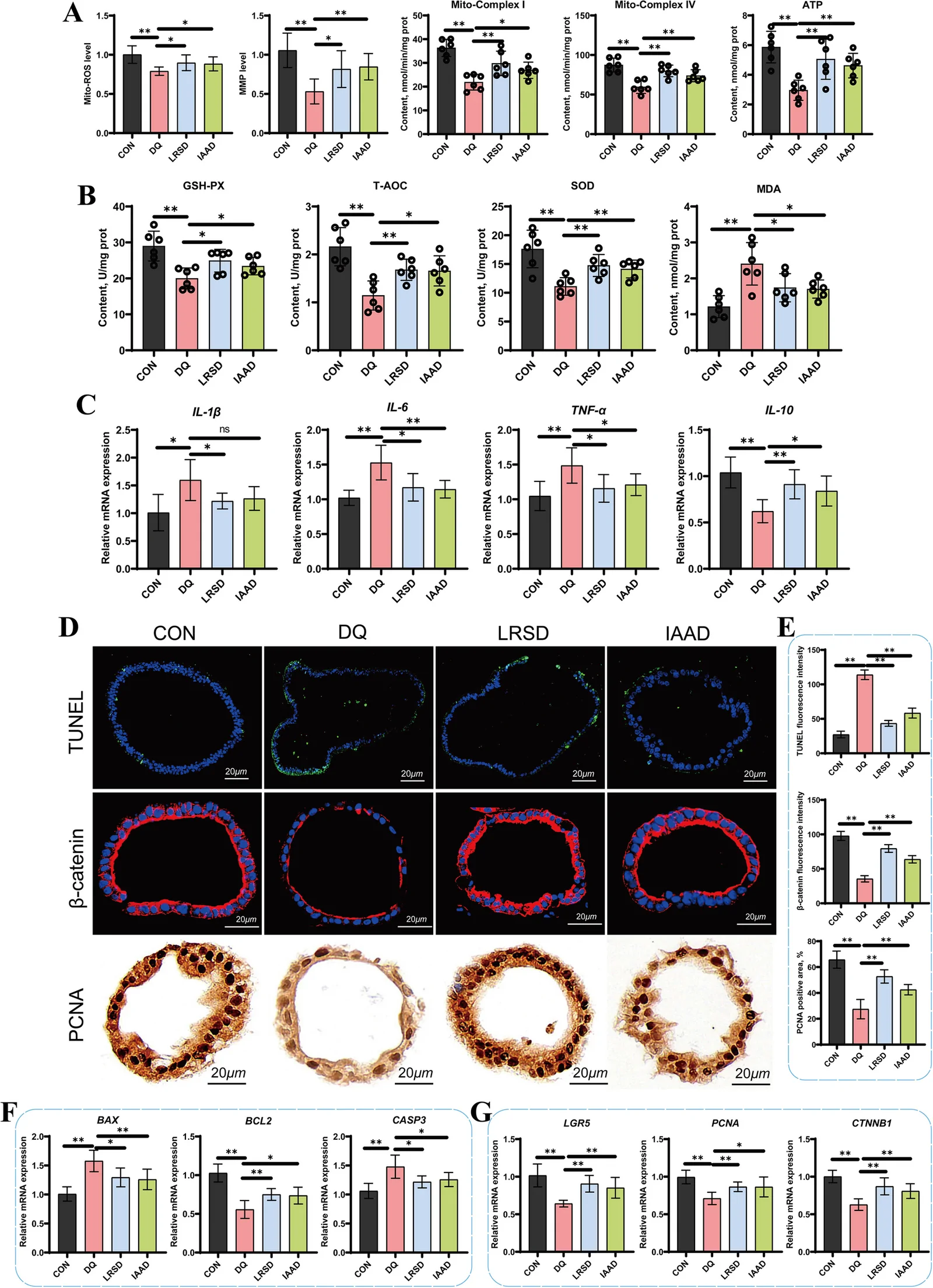
Tryptophan metabolites attenuate mitochondrial dysfunction, oxidative stress, inflammation, and apoptosis in intestinal organoids. **A** Mito-ROS level, mitochondrial membrane potential (MMP), Mito-Complex I content, Mito-Complex IV content, and ATP content. **B** Antioxidant and oxidative stress markers: GSH-PX, T-AOC, SOD, and MDA content. **C** mRNA expression of inflammatory cytokines *IL-1β*, *IL-6*, *TNF-α*, and *IL-10*. **D** Representative immunofluorescence images of TUNEL staining and β-catenin staining, and immunohistochemical images of PCNA staining in organoid. **E** Quantification of TUNEL fluorescence intensity, β-catenin fluorescence intensity, and PCNA-positive area. **F** mRNA expression of apoptosis-related genes *BAX*, *BCL2*, and *CASP3*. **G** mRNA expression of the stem cell marker *LGR5*, proliferation marker *PCNA*, and *CTNNB1*. Data are presented as mean ± SD (*n* = 6). ^*^P < 0.05, ^**^P < 0.01; ns, not significant

To investigate the direct protective effects of *L. reuteri*-derived metabolites, diquat-injured intestinal organoids were pretreated with *L. reuteri* supernatant or IAA. Compared with the CON group, DQ treatment significantly impaired mitochondrial function, including decreased MMP, respiratory chain complex I and IV activities, and ATP content, while increasing mitochondrial ROS (*P* < 0.01, Fig. 9A). Both LRSD and IAAD markedly restored MMP, complex activities, and ATP levels, and reduced ROS production (*P* < 0.05). DQ exposure also disrupted antioxidant capacity by decreasing GSH-Px, T-AOC, and SOD activities and increasing MDA levels (*P* < 0.01, Fig. 9B). These changes were significantly reversed by LRSD and IAAD treatment (*P* < 0.05). At the inflammatory level, DQ upregulated mRNA expression of pro-inflammatory cytokines (*IL-1β*, *IL-6*, *TNF-α*) and downregulated *IL-10* (*P* < 0.05, Fig. 9C). LRSD and IAAD effectively suppressed the inflammatory response (*P* < 0.05). Furthermore, DQ markedly increased TUNEL-positive apoptotic signals and decreased β-catenin and PCNA fluorescence intensity (Fig. 9D–E). Both LRSD and IAAD interventions significantly reduced apoptosis and restored β-catenin and PCNA expression. Consistent with these findings, DQ upregulated *BAX* and *CASP3* while downregulating *BCL2*, *LGR5*, *PCNA*, and *CTNNB1* at the mRNA level; these alterations were largely reversed by LRSD and IAAD (*P* < 0.05, Fig. 9F–G).

### Tryptophan metabolites restore intestinal barrier integrity and epithelial differentiation in DQ-injured intestinal organoids

**Fig. 10:**
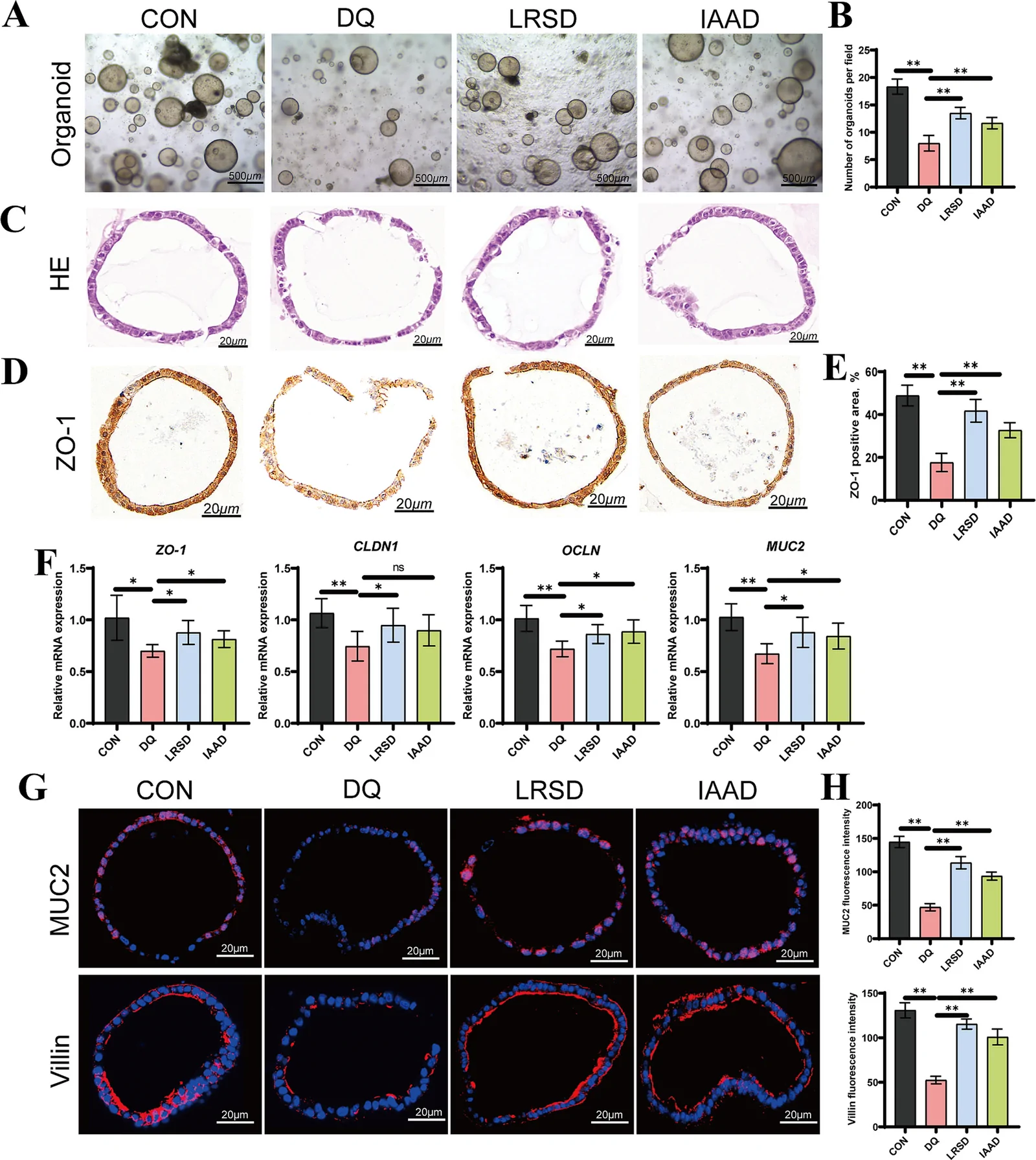
Tryptophan metabolites restore intestinal barrier integrity and epithelial differentiation in DQ-injured intestinal organoids. **A** Representative bright-field images of organoids. **B** Quantification of organoid number per field. **C** Representative HE-stained organoid cross-sections. **D** Representative immunohistochemical images of ZO-1 staining. **E** Quantification of ZO-1-positive area (%). **F** mRNA expression of the tight junction and mucin genes *ZO-1*, *CLDN1*, *OCLN*, and *MUC2*. **G** Representative immunofluorescence images of MUC2 and Villin staining in organoid cross-sections. **H** Quantification of MUC2 and Villin fluorescence intensity. Data are presented as mean ± SD (*n* = 6). ^*^P < 0.05, ^**^P < 0.01; ns, not significant

Bright-field imaging showed that the number of organoids per field was significantly reduced by DQ treatment compared with CON (*P* < 0.01, Fig. 10A and B), and this was significantly rescued by LRSD and IAAD treatment (*P* < 0.01). HE staining (Fig. 10C) revealed disrupted epithelial architecture in DQ-treated organoids, whereas LRSD and IAAD groups exhibited a more well-organized epithelium resembling that of CON. Immunohistochemistry showed that ZO-1-positive area was markedly reduced in DQ-treated organoids compared with CON (*P* < 0.01, Fig. 10D and E), and significantly increased following LRSD and IAAD treatment (*P* < 0.01). At the mRNA level (Fig. 10F), DQ treatment significantly downregulated *ZO-1*, *CLDN1*, *OCLN*, and *MUC2* expression compared with CON (*P* < 0.05). LRSD and IAAD treatment significantly restored *ZO-1*, *OCLN*, and *MUC2* expression (*P* < 0.05). Immunofluorescence staining showed that MUC2 and Villin fluorescence intensity were both markedly reduced in DQ-treated organoids compared with CON (*P* < 0.01, Fig. 10G–H). Treatment with LRSD and IAAD significantly restored both MUC2 and Villin fluorescence intensity relative to DQ (*P* < 0.01).

## Discussion

Intestinal oxidative stress represents a critical challenge in modern laying hen production, as aging, environmental and nutritional challenges trigger excessive ROS production, leading to barrier dysfunction, systemic inflammation, reduced egg production, deteriorated eggshell quality, and ultimately substantial economic losses to the poultry industry [46, 47]. Therefore, alleviating intestinal barrier damages is the principal target in poultry production. *L. reuteri*, a well-characterized probiotic with remarkable colonization capacity and metabolic versatility, has demonstrated efficacy in alleviating intestinal oxidative stress and barrier injury [48–50]. The present study investigated how *L. reuteri* alleviates diquat-induced intestinal injury in laying hens through coordinated restoration of gut microbiota homeostasis, enrichment of tryptophan metabolites, recovery of mitochondrial function, rebalancing of Nrf2-NF-κB signaling crosstalk, and ultimately restoration of intestinal barrier integrity.

Extensive studies have established that gut microbiota homeostasis is critical for maintaining intestinal barrier and metabolic regulation [51]. Previous research has consistently demonstrated that oxidative stress disrupts gut microbiota homeostasis, which are frequently accompanied by metabolic perturbations [52]. Consistently, our study observed that diquat-induced oxidative stress in laying hens disrupted cecal microbiota homeostasis, characterized by the reduced *Lactobacillus*, elevated Firmicutes/Bacteroidota ratio and enhanced harmful bacteria such as *Ruminococcus_torques_group*. This microbiota dysbiosis was accompanied by the depletion of tryptophan metabolites. Studies have showed that *L. reuteri* can restore gut microbiota dysbiosis and modulates metabolic profiles in various intestinal inflammation and oxidative stress models [53]. Our study also found that *L. reuteri* pretreatment effectively restored the microbiota and metabolism in our diquat-challenged laying hens. Specifically, *L. reuter* increased the abundance of *Lactobacillus*, which is often related to intestinal barrier restoration. *Lactobacillus* has been reported to encourage intestinal epithelium regeneration [54], regulate tight junction protein [55] and maintain mucus layer thickness [56]. Meanwhile, our results also show that tryptophan metabolism is the top upregulated pathway, with multiple metabolites significantly elevated, including IAA, *trans*-3-indoleacrylic acid and indole-3-aldehyde. Tryptophan metabolism is highly correlated with intestinal health [57]. Previous studies have demonstrated that indole metabolites alleviate intestinal barrier damage, promote epithelial repair, and inhibit inflammation [58]. Tryptophan metabolites can also regulate the expression of tight junction proteins to mitigate intestinal barrier [59]. In the intestinal organoid validation experiments, both *L. reuteri* supernatant and IAA significantly alleviated inflammation and improved tight junction integrity, confirming the direct protective role of *L. reuteri*-derived metabolites on the intestinal epithelium. Thus, the interaction of *Lactobacillus reuteri* with microbiota and metabolism may facilitate the intestinal barrier recovery.

Mitochondria function both as major ROS generators and as primary targets of ROS-induced damage in oxidative stress pathophysiology [60]. Mitochondria dysfunction may hinder intestinal epithelium function, leading to intestinal barrier damage [61]. Studies have shown that *Lactobacillus reuteri* can mitigate oxidative stress, alleviate mitochondria dysfunction [62, 63] and improve intestinal health [64, 65]. Consistently, in our study *L. reuteri* intervention reduces ROS production and restores mitochondrial integrity both in jejunal tissues and organoids. This may be because tryptophan metabolites of *Lactobacillus reuteri* improve mitochondrial function, reduce ROS accumulation, and protect mitochondrial integrity [66]. Meantime, we found *L. reuteri* recover ΔΨm and ATP content. Mitochondrial ATP production is essential for intestinal barrier maintenance, as they facilitate tight junction integrity, as well as influencing the expression of *MUC2* [67]. Importantly, in the organoid model, both LRSD and IAAD significantly improved mitochondrial respiratory chain complex activities and restored ATP levels, providing direct evidence that *L. reuteri*-derived metabolites can protect mitochondrial function. Our study also observed upregulated PGC-1α related biogenesis genes, rebalanced fission and fusion dynamics and normalized mitophagy. This may be because *L. reuteri’s* tryptophan metabolites, such as IAA, can boost mitochondrial biosynthesis, reduce oxidative stress [68], and promote a healthy mitophagy to maintain mitochondrial function [14]. Thus, upregulated PGC-1α promotes intestinal epithelial differentiation and mitochondrial biogenesis [69], rebalanced mitochondrial dynamics alleviates inflammation and epithelial damage [70] and moderate mitophagy clears damaged mitochondria and protect the intestinal barrier [71]. *L. reuteri* strongly regulates these functional pathways and restores mitochondrial health, thereby promoting intestinal barrier integrity.

The Nrf2 and NF-κB pathways represent two master pathways that determine cellular responses to oxidative stress [72]. Notably, the Nrf2/HO-1 antioxidant pathway and TLR4/NF-κB inflammatory pathway are not independent but exhibit significant crosstalk under oxidative stress. Excessive ROS acts as a central upstream signal that simultaneously inhibits Nrf2 nuclear translocation (by promoting Keap1 activity or GSK-3β-mediated Nrf2 degradation) and activates TLR4/NF-κB signaling through IKK-mediated IκB degradation [73]. In turn, overactivated NF-κB can further suppress Nrf2 transcriptional activity by competing for limited transcriptional co-activators (e.g., CBP/p300) and by upregulating Keap1 expression, thereby forming a self-reinforcing oxidative-inflammatory vicious cycle [74, 75]. Nrf2 maintains the proliferation balance of intestinal stem cells [76] and NF-κB promotes apoptosis [77]. They collectively regulate downstream apoptosis and proliferation. Previous studies have shown that oxidative stress, while inhibiting Nrf2-mediated antioxidant defense, overactivates NF-κB-driven inflammatory responses [78], promotes apoptosis rather than proliferation, and ultimately leads to intestinal epithelial cell damage and barrier dysfunction [79]. Extensive evidence has demonstrated that *L. reuteri* alleviates intestinal damage by activating the Nrf2/HO-1 pathway [80], while inhibiting the NF-κB pathway to exert anti-inflammatory effects [81]. *L. reuteri* also improved the expression of intestinal TJs and maintained the integrity of the intestinal barrier by inhibiting apoptosis of intestinal epithelial cells [82] and stimulating the expansion of intestinal stem cells [83]. Consistently, *L. reuteri* intervention in our study rebalanced this molecular crosstalk by enhancing Nrf2 antioxidant defenses and suppressing NF-κB inflammatory signaling pathway. Furthermore, *L. reuteri* attenuated apoptosis, promoted proliferation and restored intestinal barrier. In our organoid validation study, *L. reuteri* supernatant and IAA effectively enhanced antioxidant enzyme activities, suppressed inflammatory genes expression, inhibited apoptosis-related proteins, and promoted proliferation markers, further supporting the protective role of *L. reuteri* metabolites on oxidative-inflammatory balance and epithelial renewal. Mechanistically, *Lactobacillus reuteri* may influence upstream tryptophan metabolites to improve the oxidative-inflammatory balance, thereby alleviating intestinal damage. Studies have shown that tryptophan metabolites such as IAA and ILA can activate AhR/Nrf2 signaling to promote gut barrier [84], alleviate intestinal epithelial cell injury via regulation of the TLR4/NF-κB pathway to reduce mucosal damage and apoptosis [85], and modulate the proliferation of intestinal epithelial cell [86]. Meanwhile, restored mitochondrial function reduced ROS generation at the source, alleviating the oxidative trigger for both Nrf2 dissociation inhibition and TLR4-mediated NF-κB activation [87]. Finally, increased anti-inflammatory cytokine and restored mitochondrial function decreased intrinsic apoptosis signaling [88], while Nrf2 activation supported Wnt/β-catenin signaling to maintain intestinal stem cell populations and promote epithelial renewal.

The present study has several limitations. First, although six replicate pens were used per treatment, only one hen per replicate was sampled for most analyses. This experimental design may have reduced statistical power. Second, although we observed activation of the Nrf2/HO-1 antioxidant pathway and suppression of the TLR4/NF-κB inflammatory pathway in vivo experiments, both pathways were not validated in the intestinal organoid model. Given that oxidative stress is the core theme of this study, direct intervention on the Nrf2/HO-1 antioxidant pathway would have provided stronger mechanistic insight. Third, while the organoid validation experiments demonstrated that *L. reuteri* supernatant and IAA could improve mitochondrial function, antioxidant capacity, and reduce inflammation and apoptosis, they mainly assessed phenotypic indicators and did not deeply investigate the effects on mitochondrial biogenesis, dynamics balance, or mitophagy regulation. Future studies incorporating targeted pathway interventions and more comprehensive mechanistic validation are necessary to fully elucidate the causal relationships.

## Conclusion

**Fig. 11:**
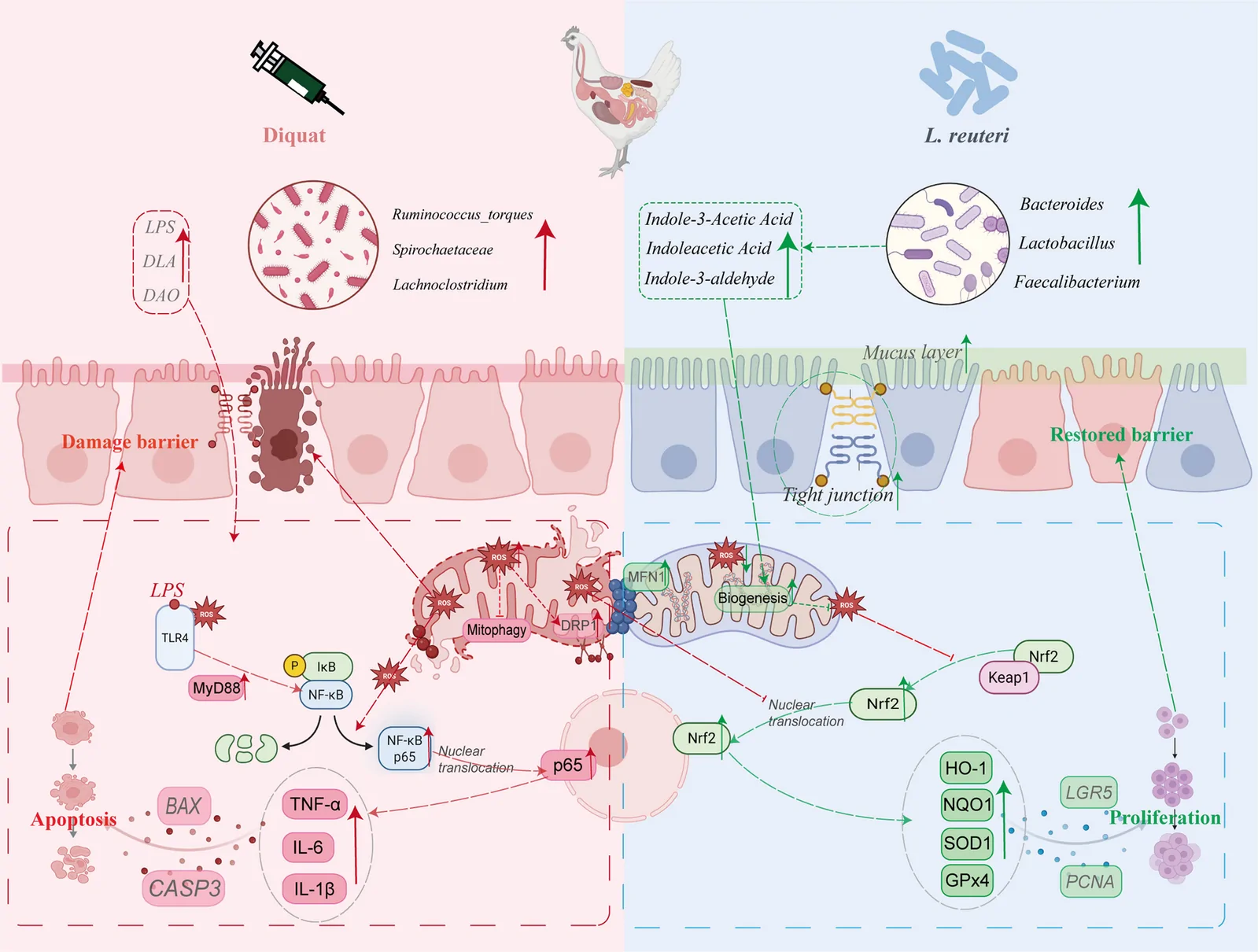
Mechanism by which *Limosilactobacillus reuteri* alleviates diquat-induced intestinal oxidative stress injury in laying hens.

In conclusion, this study unveils the mechanisms that *L. reuteri* alleviates intestinal oxidative damage through coordinated regulation of gut microbiota, tryptophan metabolite profiles, mitochondrial function, and stem cell renewal, thereby reducing oxidative stress and enhancing intestinal barrier integrity (Fig. 11). These findings not only advance our fundamental understanding of probiotic mechanisms but also provide a rational foundation for developing microbiome-targeted nutritional interventions to enhance intestinal health and productive performance in laying hens under oxidative stress conditions.

## Supplementary Information

Supplementary Material 1: Table S1. Effects of *L. reuteri* on the production performance of older laying hens. Table S2. Laying rate on d 1–7 post-injection of diquat. Table S3. Primers used for quantitative real-time PCR.

Supplementary Material 2. Probiotic properties and survival rate in various stimulation of *Limosilactobacillus reuteri*.

Supplementary Material 3. WB images supplementary file.

## Data Availability

The data used or analysed during the current study are available from the corresponding author on reasonable request.

## Abbreviations

4-HNE: 4-Hydroxynonenal
ADEM: Average daily egg mass
ADFI: Average daily feed intake
CTNNB1: Catenin beta 1
BCL-2: B-cell lymphoma-2
CAS-3: Cysteinyl aspartate specific proteinase-3
CD: Crypt depth
CLDN1: Claudin1
DAO: Diamine oxidase
D-LA: D-Lactic acid
DQ: Diquat
DRP1: Dynamin-related protein 1
FCR: Feed conversion ratio
FIS1: Fission 1
GPX-4: Glutathione peroxidase-4
GSH-PX/GPX: Glutathione peroxidase
HO-1: Heme oxygenase-1
IκBα: Inhibitor of kappa B alpha
IL-1β: Interleukin-1β
IL-6: Interleukin-6
IL-10: Interleukin-10
Keap-1: Kelch-like ECH-associated protein-1
LC3B: Microtubule-associated protein 1 light chain 3B
LGR5: Leucine-rich repeat-containing G-protein coupled receptor 5
LPS: Lipopolysaccharide
LR: Laying rate
MDA: Malondialdehyde
MFN1: Mitofusin 1
MFN2: Mitofusin 2
MMP: Mitochondrial membrane potential
mtDNA: Mitochondrial DNA
MUC2: Mucin-2
MyD88: Myeloid differentiation primary response 88
NF-κB: Nuclear factor kappa B
NF-κB p-p65: Phosphorylated p65 subunit of NF-κB
NQO-1: NAD(P)H:quinone oxidoreductase-1
Nrf2: Nuclear factor erythroid 2-related factor 2
NRF1: Nuclear respiratory factor 1
OCLN: Occludin
Parkin: E3 ubiquitin-protein ligase
PCNA: Proliferating cell nuclear antigen
PINK1: PTEN-induced kinase 1
PGC-1α: Peroxisome proliferator-activated receptor gamma coactivator 1-alpha
POLRMT: Mitochondrial RNA polymerase
SOD: Superoxide dismutase
T-AOC: Total antioxidant capacity
TFAM: Transcription factor A, mitochondrial
TLR4: Toll-like receptor 4
TNF-α: Tumor necrosis factor alpha
TUNEL: Terminal deoxynucleotidyl transferase dUTP nick end labeling
VH: Villus height
ZO-1: Zonula occludens
