# Supplementary material for: Limosilactobacillus reuteri orchestrates IAA-mediated mitochondrial homeostasis and stem cell renewal against intestinal oxidative injury in aged hens
Source: J Anim Sci Biotechnol. 2026 Sep 14;17:184. doi: 10.1186/s40104-026-01499-4 (PMC13573404; doi:10.1186/s40104-026-01499-4)
Supplement: Supplementary file 1 [file 40104_2026_1499_MOESM1_ESM.docx]

**Supplementary material 1**

*Limosilactobacillus reuteri* orchestrates IAA-mediated mitochondrial homeostasis and stem cell renewal against intestinal oxidative injury in aged hens

**Table S1** Effects of *L. reuteri* on the production performance of older laying hens

| **Item** | **CON** | **LR** | ***P-value*** |
| --- | --- | --- | --- |
| Laying rate, % | |  |  |
| Week 71-75 | 79.80±0.95^b^ | 83.40±0.63^a^ | <0.001 |
| Week 76-80 | 76.60±1.11^b^ | 81.20±0.79^a^ | 0.018 |
| Week 71-80 | 78.20±1.95^b^ | 82.30±1.34^a^ | <0.001 |
| Average daily egg weight，g | |  |  |
| Week 71-75 | 51.23±0.48^b^ | 53.75±0.28^a^ | <0.001 |
| Week 76-80 | 49.50±0.62^b^ | 52.71±0.40^b^ | 0.021 |
| Week 71-80 | 50.37±1.05^b^ | 53.23±0.64^a^ | 0.004 |
| Average daily feed intake, g/hen per day | |  |  |
| Week 71-75 | 114.53±0.64 | 113.88±1.03 | 0.587 |
| Week 76-80 | 112.49±1.12 | 113.98±1.10 | 0.167 |
| Week 71-80 | 115.51±1.41 | 113.93±1.01 | 0.873 |
| Feed conversion ratio, g/g | |  |  |
| Week 71-75 | 2.23±0.03^a^ | 2.12±0.03^b^ | <0.001 |
| Week 76-80 | 2.27±0.04^a^ | 2.16±0.06^b^ | 0.025 |
| Week 71-80 | 2.25±0.04^a^ | 2.14±0.05^b^ | 0.016 |

Note: Data are mean ± standard error. Different superscripts in the same row indicate significant differences between groups (*P* < 0.05), while identical letters or no letters indicate no significant differences (*P* > 0.05). (*n* = 6)

**Table S2** Laying rate on days 1–7 post-injection of diquat

| Days (post-injection) | CON | DQ | LRD | *P*-value |
| --- | --- | --- | --- | --- |
| Day1 | 75.81 ± 1.35^a^ | 14.57 ± 1.43^c^ | 25.17 ± 1.21^b^ | <0.001 |
| Day2 | 75.43 ± 1.07^a^ | 20.81 ± 1.50^b^ | 33.53 ± 1.29^ab^ | <0.001 |
| Day3 | 75.64 ± 1.01^a^ | 26.26 ± 1.72^c^ | 41.82 ± 1.37^b^ | <0.001 |
| Day4 | 74.93 ± 1.22^a^ | 33.53 ± 1.80^c^ | 49.76 ± 1.52^b^ | <0.001 |
| Day5 | 75.37 ± 1.09^a^ | 32.79 ± 2.06^c^ | 48.91 ± 1.61^b^ | <0.001 |
| Day6 | 74.70 ± 1.34^a^ | 34.09 ± 1.94^c^ | 50.13 ± 1.42^b^ | <0.001 |
| Day7 | 75.15 ± 1.24^a^ | 35.27 ± 1.76^c^ | 51.06 ± 1.49^b^ | <0.001 |
| Average | 75.29 ± 0.39^a^ | 28.19 ± 7.94^c^ | 42.91 ± 10.04^b^ | <0.001 |

Note: Data are mean ± standard error. Different superscripts in the same row indicate significant differences between groups (*P* < 0.05), while identical letters or no letters indicate no significant differences (*P* > 0.05). (*n* = 6)

**Table S3** Primers used for quantitative real-time PCR

| Gene | Primer Sequence (5’ to 3’) | Accession No. |
| --- | --- | --- |
| *MFN1* | F: AGTTCTGCCGGACACTGAAG | XM_015291425.4 |
|  | R: CCTCCGCCATGATGTAACGAA |  |
| *MFN2* | F: ACTTCCATGGGGATCATCGT | XM_015882356.1 |
|  | R: GGCGTTCATACACATAAAGCAG |  |
| *DRP1* | F: TGGGCACGCAGAGCAG | NM_001079722.2 |
|  | R: AGCATCTATCTCATTTTCATCTCCA |  |
| *FIS1* | F: GGACGACCTGCTGAAGTTTG  R: CGTTGTACTTGCTTCGCACC | XM_006773401.2 |
|  |  |  |
| *LGR5* | F: TACGTCTTGCAGGAAATGGCT  R: GGAACCTGGCGTAGTTGGTTA | XM_046909876.1 |
| *β-catenin* | F: TGCGAATCAACCCAACAGTA  R: CTCACCAGCAGACATCAGGA | NM_205081.3 |
| *PCNA* | F: CAGATGTTCCTCTCGTTGTGGA  R: AGCAGATCACCCCCTCAGTC | NM_204170.3 |
| *Claudin-1* | F: TGGCCACGTCATGGTATGG  R: AACGGGTGTGAAAGGGTCATAG | NM_001013611.2 |
| *ZO-1* | F: TATGAAGATCGTGCGCCTCC | XM_015278981.1 |
|  | R: GAGGTCTGCCATCGTAGCTC |  |
| *Occludin* | F: TCATCGCCTCCATCGTCTAC | NM_205128.1 |
|  | R: TCTTACTGCGCGTCTTCTGG |  |
| *MUC-2* | F: AGCGAGATGT TGGCGATGAT | NM_001318434.1 |
|  | R: AAGTTGCCACACAGACCACA |  |
| *Caspase‑3* | F: TCCACCGAGATACCGGACTG | NM_204725.1 |
|  | R: ACAAAACTGCTTCGCTTGCT |  |
| *Bax* | F: GGGGTACGTCAATGTGGTCA | NM_001030920.1 |
|  | R: AGGAAGGCGGTGGGATAATG |  |
| *Bcl‑2* | F: ATGACCGAGTACCTGAACCG | NM_205339.2 |
|  | R: CAAGAGTGATGCAAGCTCCC |  |
| *mtD-loop* | F: AGGACTACGGCTTGAAAAGC | MT800504.1 |
|  | R: CATCTTGGCATCTTCAGTGCC |  |
| *NRF1* | F: ACACAGCAACAGACCACAAC | NM_001030646.1 |
|  | R: AACCTGGATGAGGGACACAG |  |
| *TFAM* | F: GAAACGTGGCAAAATCTATCCG | XM_015866188.1 |
|  | R: AGGTCTTCGCGTCCAAGCTC |  |
| *PGC-1α* | F: CATGTGCAACCAGGACTCTGT | NM_001006457.1 |
|  | R: ACGTCTAGTTCGGAGAGGTCA |  |
| *POLRMT* | F: CTTTGAGGTTTTCCAGCAGCAG  R: CTTTGAGGTTTTCCAGCAGCAG | XM 001927064.1 |
| *Nrf2* | F: CAGGGGTAGCAAGGTATGAGG  R: TTCCCAGTTCGGTGCAGAAG | NM_205117.2 |
| *Keap1* | F: TCAACTGGGTGCAGTACGAC  R: TCTGCGCCAGGTAATCCTTG | MN416132.1 |
| *HO-1* | F: CCACACAACGCTGAAAGCAT  R: GATGAAGTACAGGGACGCCG | NM_205344.2 |
| *NQO1* | F: AACCTCTTTCAACCACGCCA  R: AAGCACTCGGGGTTCTTGAG | NM_001277619.2 |
| *SOD1* | F: TGACCTCGGCAATGTGACTG  R: CATGGTACGGCCAATGATGC | NM_205064.2 |
| *GPX4* | F: ACGCTGGGGAATGCCATCA  R: CGGGCAGATCCTTCTCGATCA | NM_001346448.2 |
| *IL-1β* | F: TGCCTGCAGAAGAAGCCTCG | NM_204524.1 |
|  | R: CTCCGCAGCAGTTTGGTCAT |  |
| *IL-6* | F: GCAGGACGAGATGTGCAAGA | NM_204628.1 |
|  | R: ACCTTGGGCAGGTTGAGGTT |  |
| *IL-10* | F: GTCACCGCTTCTTCACCTGC | NM_001004414.2 |
|  | R: TCCCGTTCTCATCCATCTTCTCG |  |
| *TNF-α* | F: CCCTACCCTGTCCCACAACC | XM_046927265.1 |
|  | R: TGGGCGGTCATAGAACAGCA |  |
| *IκBα* | F: CTTCCAGAACAACCTCAGCCAGAC | NM_001001472.3 |
|  | R: CGCAGCCAGCCTTCAGCAG |  |
| *MyD88* | F: AGGATGGTGGTCGTCATTTC | NM_001030962.2 |
|  | R: TTGGTGCAAGGATTGGTGTA |  |
| *NF-κB* | F: TGGAGAAGGCTATGCAGCTT | NM_205134.1 |
|  | R: CATCCTGGACAGCAGTGAGA |  |
| *TLR4* | F: TGAAAGAGCTGGTGGAACCC | NM_001030693.2 |
|  | R: CCAGGACCGAGCAATGTCAA |  |
| *β-actin* | F: ACCGGACTGTTACCAACACC | NM_205518.1 |
|  | R: CCTGAGTCAAGCGCCAAAAG |  |
